# Whole-Chain Tick Saliva Proteins Presented on Hepatitis B Virus Capsid-Like Particles Induce High-Titered Antibodies with Neutralizing Potential

**DOI:** 10.1371/journal.pone.0136180

**Published:** 2015-09-09

**Authors:** Philipp Kolb, Reinhard Wallich, Michael Nassal

**Affiliations:** 1 University Hospital Freiburg, Internal Medicine 2 / Molecular Biology, Hugstetter Str. 55, D-79106, Freiburg, Germany; 2 University of Freiburg, Biological Faculty, Schänzlestr. 1, D-79104, Freiburg, Germany; 3 University Hospital Heidelberg, Institute of Immunology, Im Neuenheimer Feld 305, D-69120, Heidelberg, Germany; University of Kansas School of Medicine, UNITED STATES

## Abstract

Ticks are vectors for various, including pathogenic, microbes. Tick saliva contains multiple anti-host defense factors that enable ticks their bloodmeals yet also facilitate microbe transmission. Lyme disease-causing borreliae profit specifically from the broadly conserved tick histamine release factor (tHRF), and from cysteine-rich glycoproteins represented by Salp15 from *Ixodes scapularis* and Iric-1 from *Ixodes ricinus* ticks which they recruit to their outer surface protein C (OspC). Hence these tick proteins are attractive targets for anti-tick vaccines that simultaneously impair borrelia transmission. Main obstacles are the tick proteins´ immunosuppressive activities, and for Salp15 orthologs, the lack of efficient recombinant expression systems. Here, we exploited the immune-enhancing properties of hepatitis B virus core protein (HBc) derived capsid-like particles (CLPs) to generate, in *E*. *coli*, nanoparticulate vaccines presenting tHRF and, as surrogates for the barely soluble wild-type proteins, cysteine-free Salp15 and Iric-1 variants. The latter CLPs were exclusively accessible in the less sterically constrained SplitCore system. Mice immunized with tHRF CLPs mounted a strong anti-tHRF antibody response. CLPs presenting cysteine-free Salp15 and Iric-1 induced antibodies to wild-type, including glycosylated, Salp15 and Iric-1. The broadly distributed epitopes included the OspC interaction sites. *In vitro*, the anti-Salp15 antibodies interfered with OspC binding and enhanced human complement-mediated killing of Salp15 decorated borreliae. A mixture of all three CLPs induced high titered antibodies against all three targets, suggesting the feasibility of combination vaccines. These data warrant *in vivo* validation of the new candidate vaccines´ protective potential against tick infestation and Borrelia transmission.

## Introduction

Ticks are obligate hematophagous ectoparasites that are important vectors for numerous microbes, including human and veterinary pathogens. Prominent examples include tick-borne encephalitis virus, Lyme disease causing borreliae, and several protozoa [1,2]. A prime reason for the ticks´ success as vectors is the immunosuppression they induce around the bite site, providing the vectored microbes a facile entry port into the vertrebrate host. Countermeasures against host defenses are a necessity for the ticks themselves. Ixodid ("hard") ticks, including Northamerican *I*. *scapularis* and Eurasian *I*. *ricinus* as main vectors for Lyme disease causing borreliae, engorge for several days during each bloodmeal, providing ample opportunity for the host to mount such defenses. Tick saliva therefore contains a complex cocktail of factors that defuse host responses such as vasoconstriction, coagulation, complement activation, and antibody induction [3].

**Fig 1:**
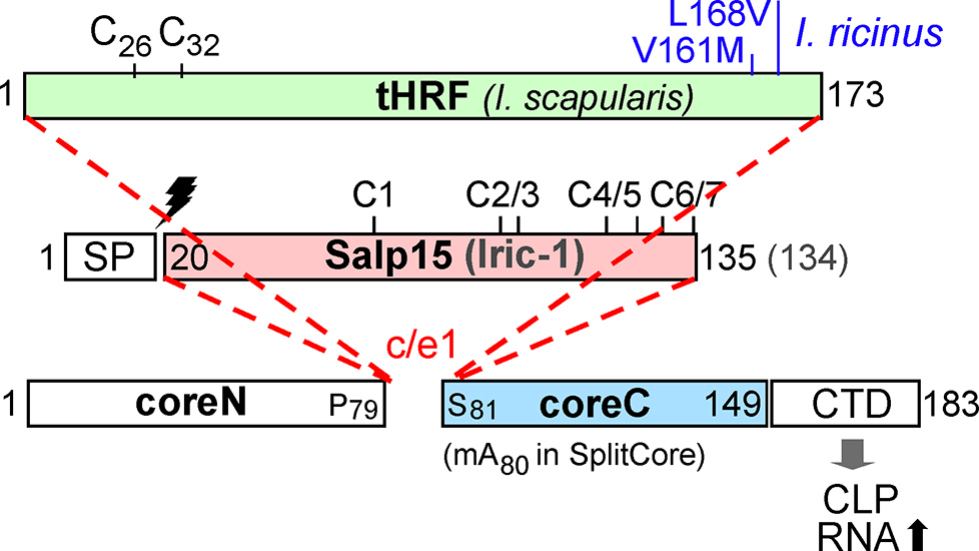
Primary sequence features of tick saliva proteins to be presented on HBc CLPs. Numbers refer to amino acid positions; the bars are drawn to scale. Amino acid exchanges in *I*. *ricinus* versus *I*. *scapularis* tHRF are indicated in blue. Salp15 and Iric-1 differ by one aa in length. In ticks, both are produced as precursors carrying a cleavable N terminal signal sequence (SP; cleavage site indicated by the lightning symbol) which was deleted in the constructs used here. The seven Cys residues are consecutively labeled C1, C2 and so forth, with the marks indicating their positions. For contiguous chain HBc fusion constructs the heterologous sequences were inserted, via short linkers, between P79 and S81; for separate expression of core N and coreC in the SplitCore system, the coreC segment starting with A80 was provided with an artificial methionine start codon (m). HBc183 constructs comprise the CTD that mediates efficient encapsidation of bacterial RNA.

One of the anti-host defense factors is the saliva protein of 15 kDa (Salp15; Fig 1), a secreted, glycosylated cysteine-rich immunosuppressive protein from *I*. *scapularis* [4]. Salp15 binds to CD4 on murine T cells [5] and to DC-SIGN on dendritic cells [6], compromising IL2 production and thus T cell proliferation. Furthermore, Salp15 is specifically recruited by *B*. *burgdorferi* to their outer surface protein C (OspC) as a protective coat against antibody-mediated killing; hence Salp15 directly facilitates Borrelia transmission [7]. Other ixodid ticks express Salp15 orthologs such as Iric-1 (Fig 1) from *I*. *ricinu*s [8]. Iric-1 shares about 75% sequence identity with Salp15 and likewise binds to OspC, including from *B*. *afzelii* and *B*. *garinii* [9,10] which together with *B*. *burgdorferi sensu stricto* represent the major Eurasian Lyme disease agents. Therefore, Salp15 and its orthologs have emerged as targets for anti-tick vaccines that may impede tick feeding *per se* and concomitantly Borrelia transmission [11–13]. Another tick saliva protein that is likely beneficial to the tick as well as vectored borreliae is tick histamine release factor (tHRF; Fig 1), a 173 aa protein of the multifunctional translationally controlled tumor protein (TCTP) superfamily [14,15] which is conserved in all eukaryotes [16], including humans. Antibodies to tHRF reportedly reduced tick feeding and *B*. *burgdorferi* transmission in mice [17]. Due to its high conservation tHRF has been proposed as target for general anti-tick vaccines [14]; for instance, tHRF from *I*. *scapularis* (Genbank accession no.: AAY66972.1) differs from the *I*. *ricinus* protein (accession no.: JAA67696.1) by only one (V161M) or two aa exchanges (V161M, L168V; our own *I*. *ricinus* isolate; RW and J. Habicht, unpublished data). However, the similarity to mammalian TCTP members, with a sequence identity of nearly 40% to human TPT1 (Genbank accession no.: CAG33317.1), may also bear a risk of inducing host auto-antibodies.

Here we set out to generate candidate vaccines against tHRF, Salp15 and Iric-1. The obvious need for overcoming the immunosuppressive properties of these proteins [5] in a vaccine was previously addressed [11,17] by using the highly potent complete Freund´s adjuvant (CFA) which is, however, unacceptable for human use. Instead we sought to present the tick antigens on a provenly immune-enhancing nanoparticulate carrier, namely capsid-like particles (CLPs) derived from hepatitis B virus (HBV) [18–20].

A fundamental practical obstacle for Salp15-based vaccines is the lack of efficient expression systems for recombinant glycosylated Salp15. Conversely, non-glycosylated Salp15 expressed in *E*. *coli* is virtually insoluble, likely due to the seven endogenous cysteine-residues. Recently we succeded in expressing Salp15 and Iric-1 as soluble fusion proteins with the bacterial DsbA protein [10] which allowed mapping of the OspC interaction site to a central region (aa 48–67) and the epitopes of two monoclonal antibodies (mabs) to two distinct overlapping sites in Salp15 (I83-N92, and D88-H99) that are highly conserved in Iric-1 [10]. Low solubility of free Salp15 and Iric-1 proteolytically released from the DsbA fusions was substantially improved when all cysteines were replaced by serines [10]. By contrast, tHRF is naturally non-glycosylated, contains only two endogenous cysteines (Fig 1), and can solubly be expressed in *E*. *coli* [17].

**Fig 2:**
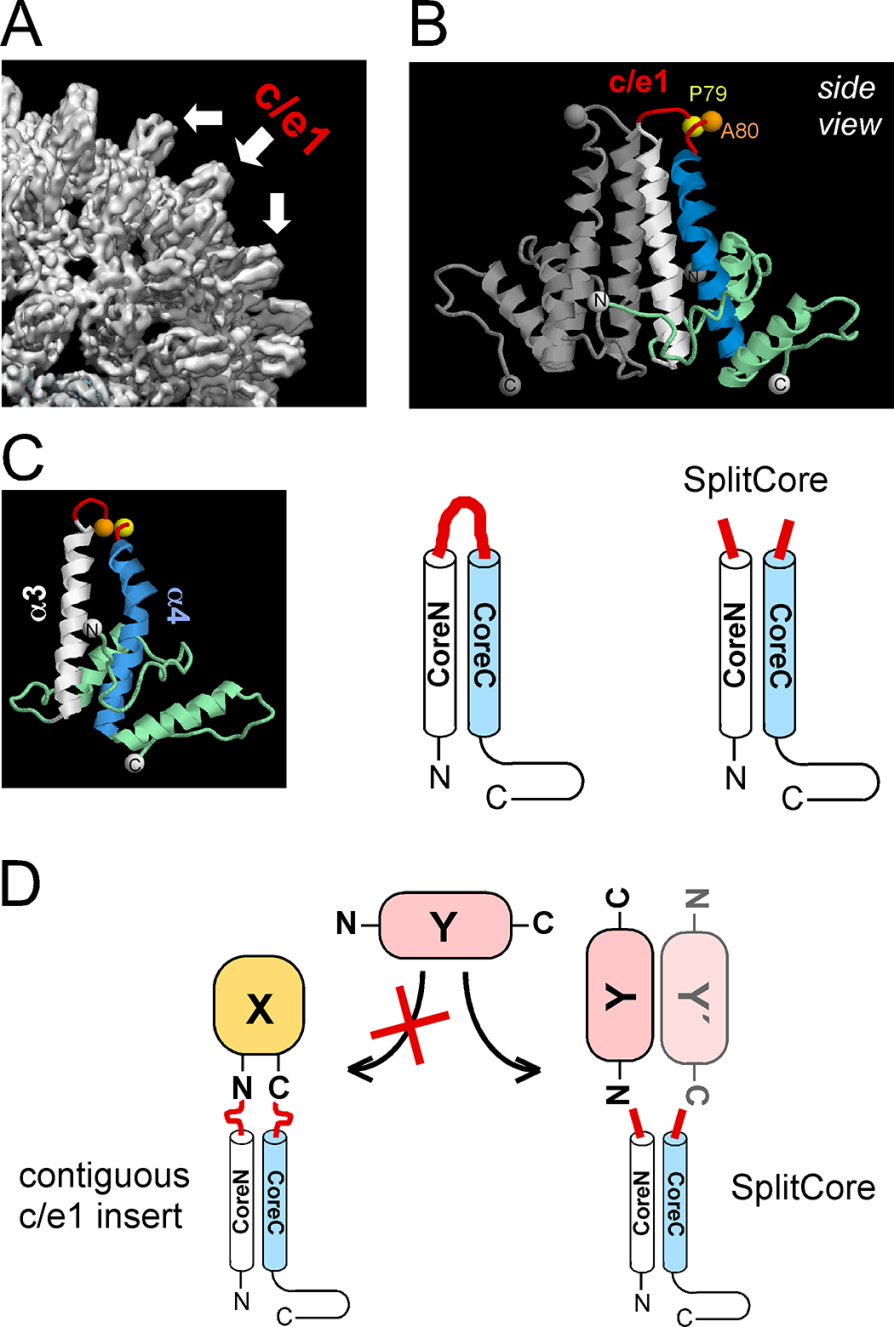
Structural features of HBc CLPs. **(A) The spikes on HBc CLPs comprise the immunodominant c/e1 epitope.** The picture shows a section from a cryo EM derived 3D reconstruction of a 120 dimer (triangulation number T = 4) HBc CLP (B. Böttcher and MN, unpublished data). **(B) X-ray derived structure of a HBc149 dimer.** The structure is based on pdb accession number 1QGT. Two core protein subunits associate into a symmetric dimer. One subunit is shown in grey, in the other the ascending central helix a3, the descending helix a4 and the connecting loop comprising the c/e1 epitope are colored white, blue and red. (**C) Correlation between the 3D structure of a HBc monomer and schematic representations of contiguous chain HBc versus SplitCore HBc. (D) HBc as carrier for heterologous proteins.** A foreign protein X with compatible 3D structure may be fused into c/e1 via both termini. A foreign protein Y with far apart termini may interfere with folding and CLP formation when fused over both ends but be compatible when fused over one end only in the SplitCore system. Fusions can be made to the coreN or the coreC segment, or both.

Virus-like particles (VLPs), genome-less surface mimics of viruses, and CLPs, derived from the capsids of enveloped viruses, are multimeric assemblies built from one or few viral protein species. Their many repetitively arrayed epitopes make them highly immunogenic [21]; their suitability as human vaccines is manifest, e.g., by the VLP vaccines against human papilloma virus infection [22]. A particularly strongly immune-enhancing nanoparticle is the capsid of HBV, an icosahedral particle [23] formed by 120 dimers (and to a lesser extent, 90 dimers) of the HBV core protein (HBc). HBc particles can act as T cell independent antigen but they also contain potent T cell epitopes [20]. The most immunogenic region is a surface-exposed loop termed c/e1 epitope (Fig 2A). It connects two long, antiparallel helices in the HBc monomer. Two such α-helical hairpins associate into a stable dimer (Fig 2B); the resulting four-helix bundles form prominent spikes on the CLP surface (Fig 2A). Of the 183 amino acids of HBc (subtype ayw; Fig 1), the N terminal 140 residues are required to assemble the capsid shell [24], whereas the highly basic C terminal domain (CTD) binds nucleic acids [24,25]. Full-length HBc as well as CTD-less variants such as HBc149 spontaneously form CLPs in *E*. *coli*; in the process, HBc183 packages the equivalent of ~3 kb of *E*. *coli* RNA [24,26], whereas HBc149 CLPs contain 10- to 20-fold less RNA [24].

Due to its exposed loop structure the c/e1 epitope lends itself for insertion of heterologous sequences. Numerous small peptide insertions did not compromise particle formation and experienced greatly increased immunogenicity [18–20]. Displaying instead whole proteins has several conceptual advantages [27], including that all epitopes of the natural antigen are presented in a genuine fashion. However, the requirement for maintaining both the carrier and the antigen in a native conformation restricts application to proteins whose structures fit into the c/e1 acceptor sites [27]; one key parameter is a close spatial juxtaposition of N and C terminus, as is the case for GFP [28] and *B*. *burgdorferi* OspC [29]; a counter example is *B*. *burgdorferi* OspA with its elongated structure [30]. This constraint has recently been remedied by a modified HBc CLP platform termed "SplitCore" [31,32]. Here the HBc molecule is physically split inside the c/e1 epitope (Fig 2C), with the two separately expressed segments efficiently associating into an assembly-competent mimic of the contiguous chain protein. A foreign protein may thus be fused to the N terminal (coreN) or C terminal (coreC) segment over one-end only and thus without the sterical constraints caused by the two-ended fixation (Fig 2D).

We therefore explored whether tHRF, Salp15 and Iric-1 could be presented on conventional or else on SplitCore CLPs and if so, whether these CLPs would induce high-titered target-specific antibodies. This was indeed easily achieved for tHRF whereas for Salp15 and Iric-1 CLP formation was restricted to the Cys-free versions and the SplitCore context. Importantly, the antibodies induced by these CLPs cross-reacted with glycosylated wild-type Salp15, covered the entire primary sequence of the target proteins and included antibodies interfering with OspC binding; furthermore, the antisera enhanced human complement-mediated killing of Salp15 decorated borreliae. Hence further evaluation of the protective potential of these new experimental vaccines against tick infestation in general and Borrelia transmission in particular appears highly warranted.

## Materials and Methods

### Expression vectors for tick proteins and HBc-tick fusions

*E*. *coli* codon optimized versions of tick protein coding sequences for *I*. *scapularis* tHRF and Salp15 and *I*. *ricinus* Iric-1 were obtained as synthetic genes from a commercial service (GeneArt). Variant Salp15 and Iric-1 genes with all endogenous Cys-residues replaced by Ser-residues were previously described [10]. A T7 promoter controlled vector for His-tagged tHRF (pET28a2-H6-tHRF) was obtained by replacing the HBc gene in vector pET28a2-HBc183 [33] by a PCR derived DNA fragment encoding the sequence MAH6SAGENLYFQGA followed by complete tHRF; for untagged tHRF (pET28a2-tHRF), the extra sequence was omitted.

pET28a2 vectors based on a synthetic core protein gene [34] encoding full-length HBc183 and C terminally truncated HBc149, without or with C terminal His6 tag, and bicistronic HBc183- and HBc149-based SplitCore constructs were previously described [31,33]. For HBc fusion proteins, the tick protein genes were PCR amplified using primers providing restriction sites compatible with sites flanking the c/e1 encoding region in the HBc gene plus short linker sequences. In contiguous chain HBc tHRF was flanked by G_5_S and G_5_ linkers, and Salp15 by G_5_ and G_4_ linkers. In SplitCore constructs, Salp15 and Iric-1 were fused to coreN via G_5_ linkers. In SplitCore REV (Salp)_2_ constructs, the second Salp cassette was fused to the N terminus of coreC via a G_3_T linker. All constructs were verified by DNA sequencing.

### Recombinant proteins

For protein expression, the appropriate pET vectors were transformed into *E*. *coli* strain BL21*CP (Stratagene) or, where indicated, in strains T7 SHexpCP (NEB) or Arctic Express (Agilent). For nonparticulate proteins, cell growth, induction with 1 mM IPTG (usally at 25°C) and cell lysis were performed as described in reference [35], for proteins expected to form CLP as described in reference [33]. H6-tHRF was purified using an ÄKTA FPLC system by Ni^2+^ IMAC and subsequent SEC on Superdex 75 (XK16/60 column; all GE Healthcare) as described for other His-tagged proteins [10,35]. For untagged tHRF the cleared bacterial lysate was loaded onto a HiTrap DEAE Fast Flow cartridge (GE Healthcare) equilibrated in TN50 buffer (25 mM Tris/HCl, 50 mM NaCl, pH 7.5) and eluted using a linear NaCl gradient (50 to 560 mM). Concentrated peak fractions were then subjected to Superdex 75 SEC. Free Iric-1 was obtained by proteolytic release from an *E*. *coli* expressed DsbA fusion protein carrying a TEV protease recognition site [10]. Secreted glycosylated Salp15 was obtained from the supernatant of HEK293 cells transfected with a cytomegalovirus (CMV) immediate early promoter driven vector [10]. Solubilization of H6-Salp15 from inclusion bodies and production of OspCa (Genbank accession no.: AAM19715.1), OspCb [29], OspC A3 and OspC YU (Genbank accession numbers: AFA85556.1 and AIK19145.1, respectively) were all done as described [10]. CLPs were obtained by sedimentation in 10% to 60% sucrose gradients [29,31,33]. Where appropriate, for further purification peak CLP fractions were dialyzed against TN300 buffer (as TN50 but containing 300 mM NaCl), concentrated by Amicon Ultrafiltration devices (50 kDa cut-off) and subjected to a second sucrose gradient sedimentation. Bacterial endotoxin was removed by extraction with Triton X114 as described [31].

### Electron microscopy

Negative staining was done using 2% uranyl acetate as previously described [36]; experiments were kindly performed by Bettina Böttcher, University of Edinburgh, UK.

### Immunoblotting

For immunoblotting, proteins were separated by SDS-PAGE, electro-blotted to polyvinylidenfluoride (PVDF) membranes (Millipore) and probed as previously described [33]. For detection of His-tagged proteins, a Tetra-His mouse mab (Qiagen) was used; Salp15 proteins were detected using mabs 19/7.4 or 18/12.1 [10]. Bound mabs were visualized using peroxidase (PO) conjugated anti-mouse antibody (Dianova) and ECL reagent (GE Healthcare), followed by exposure to X-ray film (BioMax MR, Kodak).

### Mouse immunizations

Groups of three to four Balb/c mice or C3H mice, as in references [11,17], were repeatedly immunized into the base of the tail with 10 μg of antigen per shot in a maximal volume of 100 μl in the absence or presence of 25 μl of MPL-TDM adjuvant (Sigma). Potential adjuvanting activity of HBc CLPs in *trans* was assessed by supplementation with 5 μg per shot of purified CLPs from HBc183, HBc183 coexpressed with SRPK1, or HBcCys150. Control mice were injected with PBS plus MPL. Immune sera (IS) were taken from the tail vein.

### Ethics statement

All animal experimentation was approved in advance by the Laboratory Animal Committee of the University of Heidelberg (RP Karlsruhe 35–9185.82/A-43/10) and performed in accordance with the European Community´s Council Directive of November 24, 1986 (86/609/EEC).

### Tick protein specific ELISA

Test antigens, routinely at 100 ng/well, were immobilized overnight on 96 well NUNC Maxisorp plates in coating buffer (50 mM sodium carbonate, pH 9.6). Plates were washed three times in PBST buffer (phosphate buffer saline containing 0.05% Tween-20) and blocked for 1 h at RT in blocking buffer (PBS containing 1% BSA). All additional incubations were performed in binding buffer (PBS containing 0.1% BSA) for 1 h at RT. Secreted His-tagged Salp15 derivatives from eukaryotic cell supernatants were bound to pre-blocked Ni-NTA HisSorb plates (Qiagen) as recommended by the manufacturer. Bound antibodies were detected using secondary antibody-PO conjugates with tetramethylbenzidine (TMB; Sigma) as substrate. Absorbance at 450 nm (A450nm) was monitored using a Genios Pro plate reader (Tecan). All assays were performed at least in duplicate. Mean values and standard error of the mean (SEM) as well the IS dilution giving a half-maximal signal in serial dilutions from 1:1,000 to 1:128,000 were calculated using Graphpad Prism.

### IgG subtype ratio determination

IgG subtypes of tHRF specific antibodies in IS from vaccination with c149-tHRF CLPs, c183-tHRF CLPs, neat H6-tHRF and tHRF plus MPL from day 35 post inoculation, were determined by ELISA as described above, except that in addition to non-subtype-specific anti-mouse IgG PO-conjugate (for total bound IgG) analogous assays were conducted using IgG1 and IgG2a specific secondary antibodies (Dianova). All assays were performed in triplicate.

### OspC binding competition ELISA

Sequential format assays were performed as described above, using ELISA plates coated with 100 ng per well of one of the four OspC proteins. For validation of the premix format, 13 μg of purified anti-Salp mab 18/12.1 or 19/7.4 were preincubated with 1 μg of DsbA-fused tick protein per ml reaction volume in binding buffer for 1 h at RT, and then loaded into the ELISA plate wells. IS testing in both formats was performed analogously using 2 μl of IS per reaction (1:100 final dilution). To quench cross-reactivity of the IS with *E*. *coli* components, reactions were supplemented with cleared lysate from *E*. *coli* cells expressing tHRF as a non-Salp15 related protein, using an amount containing about 5 μg of tHRF per 100 ng of Salp15. OspC—Salp15 mediated antibody binding was then monitored using secondary antibody-PO conjugates as described above.

### Impact of immunization-induced anti-Salp15 antibodies on complement-mediated killing of OspC expressing borreliae

Normal human serum (NHS) from healthy donors with no prior history of *Borrelia* spp. infection was purchased from the Heidelberg University blood bank, factor B-depleted NHS (NHS_-B_) from Complement Technology, Inc (Tyler, Texas). For heat inactivation of complement (hiNHS, hiNHS_-B_), sera were incubated at 56°C for 30 min. Serum sensitivity was assessed using *B*. *burgdorferi* isolate B313 [37,38], after confirming OspC expression, using a SYTO9 (BacLight bacterial viability kit; Molecular Probes) fluorescence-based survival assay [38]. In brief, cells grown to mid-logarithmic phase were harvested, washed and ~2.5x10^6^ spirochetes each were resuspended in 50 μl BSK-H medium supplemented with 8 μg of solubilized recombinant H6-Salp15, or BSA for control, with or without 2 μl of mouse anti-Salp15 IS or nonimmune mouse serum. After 1 h at 25°C, 50 μl of 50% NHS or hiNHS was added (final concentration 25% v/v) and incubated for 20 h at 30°C. In some experiments, NHS_-B_ or hiNHS_-B_ were employed instead. Thereafter, cells were washed in 0.9% NaCl, transferred to microtiter plates and processed as recommended by the manufacturer. Fluorescence intensities were measured in triplicate on a microtiter plate reader (Victor2 plate reader, Perkin Elmer).

### Statistical analyses

Unless indicated otherwise, mean values were determined in triplicate and analyzed by one-way ANOVA, followed by pairwise comparisons using Tukey´s post test as implemented in GraphPad Prism v. 6.02 software. P-values <0.05 were considered significant. Significance levels are indicated by *, p <0.05; **, p <0.01; and ***, p <0.001, or given as explicit p-values derived by Fisher´s least significant difference (LSD) test.

## Results

### *E*. *coli* derived tHRF is soluble, monomeric and compatible with c/e1 display on contiguous chain HBc

**Fig 3:**
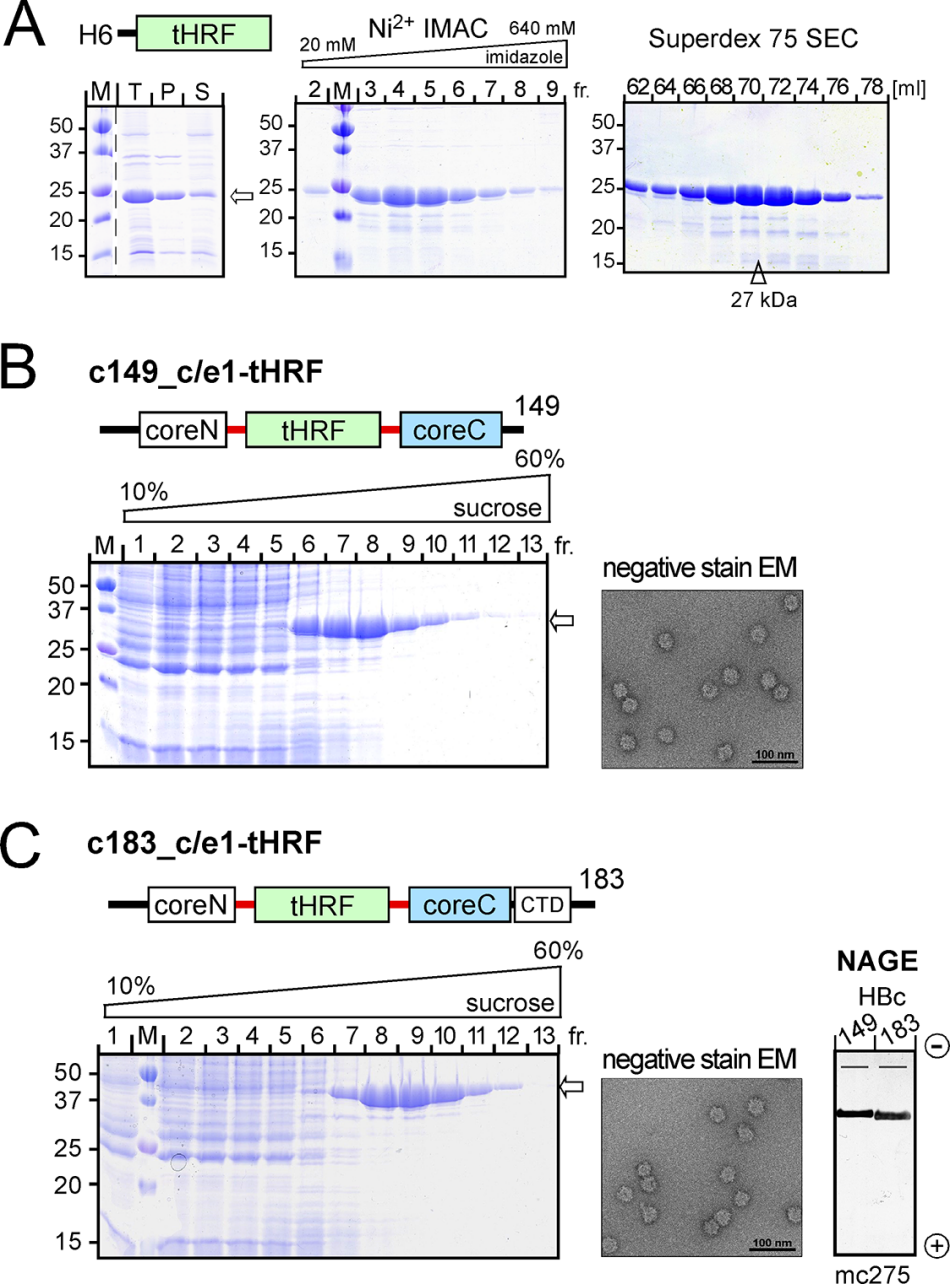
Expression in *E*. *coli* of tHRF yields soluble monomeric protein compatible with contiguous chain c/e1 insertion into HBc. **(A) N terminally His6 tagged tHRF (H6-tHRF).** *Left*: SDS-PAGE analysis of native lysate of transformed BL21*CP cells. T, total unfractionated lysate; P and S, insoluble pellet and soluble supernatant after centrifugation. The gel was stained with Coomassie Blue. *Middle*: Ni^2+^ IMAC of the soluble lysate fraction. Equal aliquots from the IMAC fractions eluted by increasing imidazole concentration were analyzed by SDS-PAGE. *Right*: Size exclusion chromatography (SEC). Peak fractions from IMAC were subjected to SEC on a Superdex S75 16/60 column; elution volumes are indicated on the top. By comparison with the elution volumes of marker proteins the peak of tHRF eluted at a volume corresponding to a molecular mass of ~27 kDa (arrowhead). This was confirmed for a non-tagged version of recombinant tHRF (S1 Fig). **(B) HBc149_c/e1-tHRF.** *Left*: Cleared native lysate of bacteria expressing the contiguous chain fusion protein were subjected to sucrose gradient sedimentation. Equal aliquots from fractions 1 to 13 (out of 14 total) were analyzed by SDS-PAGE. *Middle*: Immunoblot after native agarose gel electrophoresis (NAGE). Material from fraction 8 of the gradients shown in (B) and (C) was subjected to NAGE, blotted and detected using the HBc particle-specific mab mc275. *Right*: Negative staining EM. **(B) HBc183_c/e1-tHRF.** Sedimentation and EM analysis were performed as in (B).

As a first step towards tHRF-CLPs we generated pET-based vectors for expression of *I*. *scapularis* tHRF from an *E*. *coli* codon usage optimized synthetic gene providing an N terminal His6-tag (termed H6-tHRF). The protein was highly expressed in soluble form in *E*. *coli* BL21*CP cells and easily obtained in high yield (30 mg/L culture) and purity (>95% as judged by densitometric scanning after SDS-PAGE and Coomassie-Blue staining) by Ni^2+^ immobilized metal ion chromatography (IMAC) and subsequent size exclusion chromatography (SEC; Fig 3A). By comparison with marker proteins of known mass, H6-tHRF eluted at a volume corresponding to ~27 kDa, only slightly higher than the calculated mass of 22 kDa, in line with a monomeric state. This was not caused by the His6-tag as the same was seen with untagged tHRF purified via ion exchange chromatography and SEC (S1 Fig).

As anticipated from these favorable expression characteristics fusion constructs carrying tHRF in the c/e1 loop of HBc149-H6 and HBc183-H6 were very well expressed, mostly soluble and accumulated in the CLP-typical center fractions upon sucrose gradient sedimentation (Fig 3B and 3C). Negative staining electron microscopy (EM) revealed for both preparations abundant regular particles of about 45 nm diameter (Fig 3B and 3C); some carried discernable spikes, as expected if the displayed proteins adopt a distinct stable structure. HBc CLP typical structural features were independently confirmed by the distinct bands produced by both preparations upon native agarose gel electrophoresis (NAGE) and subsequent immunoblotting (Fig 3C) with the HBc particle-specific mab mc275 [31].

### CLP-mediated antibody response enhancement requires physical linkage to the protein antigen

**Fig 4:**
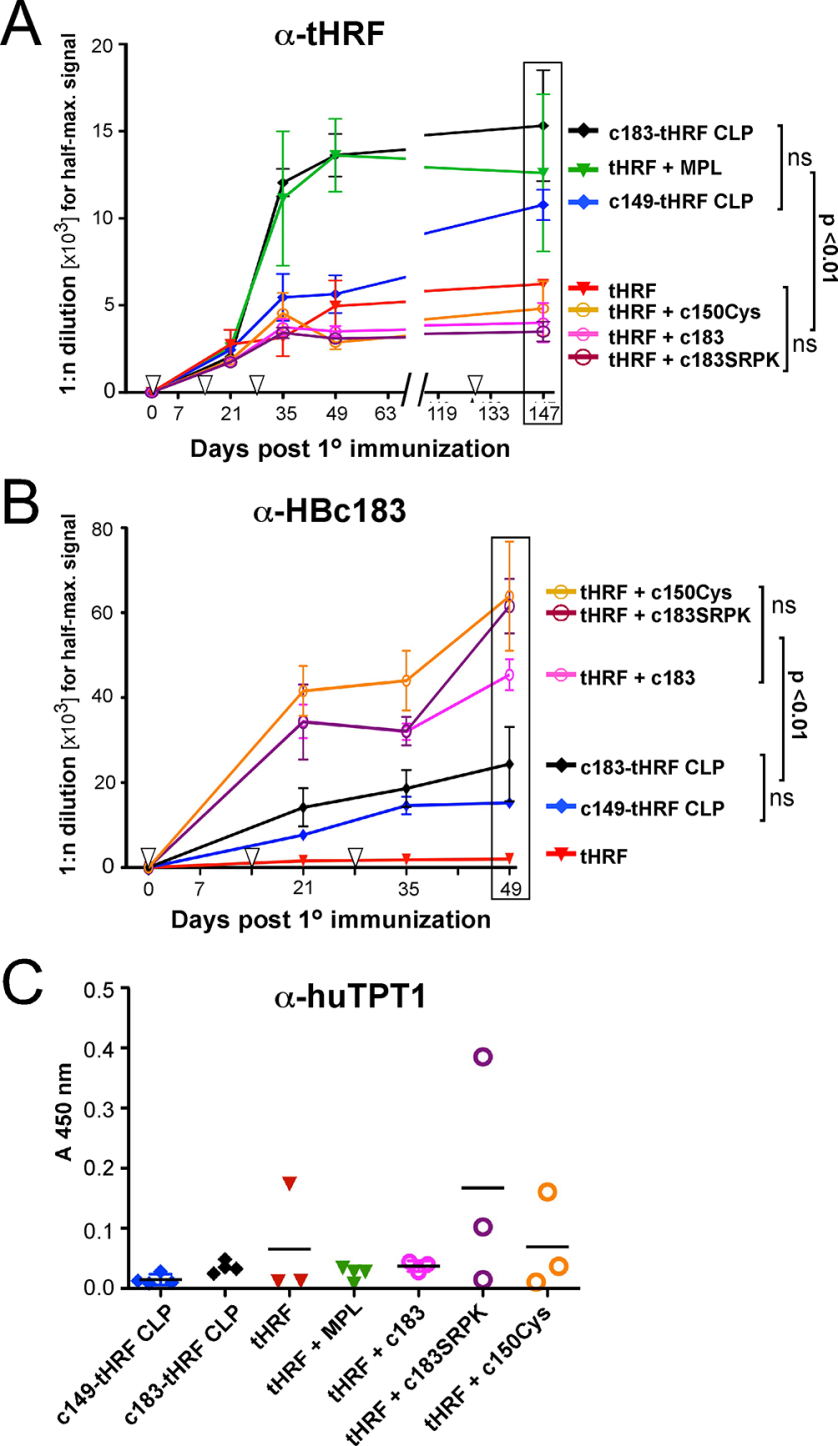
Characterization of antibodies in mouse immune sera (IS) after vaccination with different tHRF antigen preparations. Groups of three to four mice each were vaccinated at days 0, 14, 28 and 126 (downward pointing open arrowheads) with the indicated vaccine formulations. Two-fold serial dilutions of blood samples from individual mice taken at the indicated timepoints post first injection were analyzed by ELISA. The means ± SEM are plotted against time. Significance of differences between groups was assessed for the boxed time-points. **(A) Anti-tHRF response.** Dose-response curves against plate-immobilized tHRF from 1:1,000 to 1:128,000 dilutions of the individual IS from each group were used to derive the dilution giving the half-maximal ELISA reading, using Graphpad Prism software. Note that HBc CLPs administered in trans did not increase the anti-tHRF response compared to tHRF alone. **(B) Anti-HBc183 response.** Data were obtained as in (A), except HBc183 CLPs was used as immobilized test antigen. Note the high-titered anti-HBc response in all IS derived from co-administered HBc CLPs. **(C) Anti-TPT1 response.** Mouse IS from day 49 p.i. were analyzed using recombinant human TPT1 as ELISA test antigen. Due to substantial inter-animal variation, a scatter blot for the individual animals from each group is shown; the horizontal bars indicate the mean values. Note that at least one mouse from the groups immunized with tHRF alone, or tHRF plus HBc183 or HBc150Cys CLPs developed a detectable anti-TPT1 titer.

To assess their immunogenicity, the HBc149- and HBc183-based tHRF CLPs were used to vaccinate mice and compared with free H6-tHRF and H6-tHRF administered with MPL-TDM adjuvant (Sigma; hereafter shortly termed MPL); MPL (monophosphoryl-lipid A) addresses Toll-like receptor 4 (TLR4) and TDM (trehalose dicorynomycolate) the C-type lectin Mincle [39]; both are ingredients in adjuvants approved for, or in clinical trials for, use in humans [40]. In addition, we addressed a potential adjuvanting activity of three kinds of HBc CLPs co-administered with (rather than genetically linked to) H6-tHRF, namely wil-type (wt) HBc183 CLPs containing ~3 kb bacterial RNA per particle [24,26] which can act as a TLR7 agonist [41–43]; HBc183 co-expressed with serine-arginine rich protein kinase 1 (SRPK1) which phosphorylates the Arg-rich CTD of HBc [44] and strongly reduces *E*. *coli* RNA packaging ([26], and M. Vogel, J. Heger, B. Böttcher and MN, unpublished data); and HBc-Cys150 in which the entire CTD is replaced by a Cys-residue [45]. To this end, groups of 3 to 4 Balb/C mice each were repeatedly immunized (see Fig 4) with 10 μg/shot of HBc183-tHRF CLPs or HBc149-tHRF CLPs, or H6-tHRF without or with MPL; in addition, H6-tHRF was coadministered with 5 μg/shot of one of the three kinds of HBc CLPs.

Induction of specific antibodies against tHRF and HBc over time was assessed by ELISA, using microtiter plates coated with recombinant H6-tHRF or HBc183 CLPs. The wells were incubated (in duplicate) with two-fold dilution series (from 1:1,000 to 1:128,000) of immune sera (IS) from each individual mouse obtained at the indicated days post first immunization (day 0); preimmune sera (obtained on day 0 immediately prior to immunization) served as negative control. Next, the relative dose-response curves were used to determine the dilution giving the half-maximal signal (Graphpad Prism). The mean values ± SEM, derived from all individual animals from each group, are shown in Fig 4A and 4B.

In the anti-tHRF ELISA, at day 49 the highest antibody titers were detected in the IS raised against neat HBc183-tHRF CLPs and MPL-adjuvanted H6-tHRF, followed by neat HBc149-tHRF CLPs and finally by H6-tHRF alone. Samples from day 147, obtained after a final boost at day 126, showed a similar pattern, exept that titers from the HBc149-tHRF immunization increased to about two thirds the value of the anti-HBc183-tHRF CLP IS. In contrast, none of the HBc CLPs coadministered with H6-tHRF had a significant positive impact on the anti-tHRF titers (Fig 4A) as compared to H6-tHRF alone.

In the anti-HBc ELISA, sera from all mice immunized with HBc CLPs, but not those immunized with H6-tHRF alone, contained HBc-specific antibodies (Fig 4B); however, the anti-HBc titers induced by the tHRF-CLPs were all lower than those evoked by the coadministered HBc-only CLPs, in line with previous data [31].

A comparison of the IgG subtype distribution in the day 35 IS induced by HBc183-tHRF CLPs versus HBc149-tHRF-CLPs and H6-tHRF without or with MPL (S2 Fig) revealed predominantly IgG2a for the HBc183-based CLPs with high RNA content (IgG1:IgG2a ratio ≈ 1:3) yet predominantly IgG1 for the other, low RNA content samples (IgG1:IgG2a ≈ 10:1), indicating a T_H_1 promoting impact of the packaged *E*. *coli* RNA.

Lastly, we assessed potential cross-reactivity of the anti-tHRF IS against immobilized human TPT1 (Abcam). No cross-reactivity was observed for the IS induced by the tHRF-CLPs yet, notably, at least one out of three mice from the groups immunized with H6-tHRF alone or H6-tHRF plus the low RNA HBc CLPs showed evidence of TPT1 crossreactive antibodies (Fig 4C). Together, the results corroborated a strong immune-enhancing effect of displaying tHRF on HBc CLPs and demonstrated its strict dependence on a physical linkage.

### Assessment of different carrier architectures for CLP display of Salp15 and Iric-1

The aggregation tendency of Salp15 and Iric-1 might even be aggravated by bringing multiple copies into close proximity on the CLP surface. Given these poor preconditions for CLP formation, we chose to experimentally explore whether the contiguous chain HBc system or the SplitCore system would allow to generate Salp15 and/or Iric-1 presenting CLPs. To possibly counteract disulfide-mediated solubility problems, we also included the Cys-free variants of Salp15 and Iric-1. All tick proteins were used in their mature forms of about 115 aa in length, i.e. lacking the predicted N terminal signal sequences of about 20 aa (see Fig 1).

**Fig 5:**
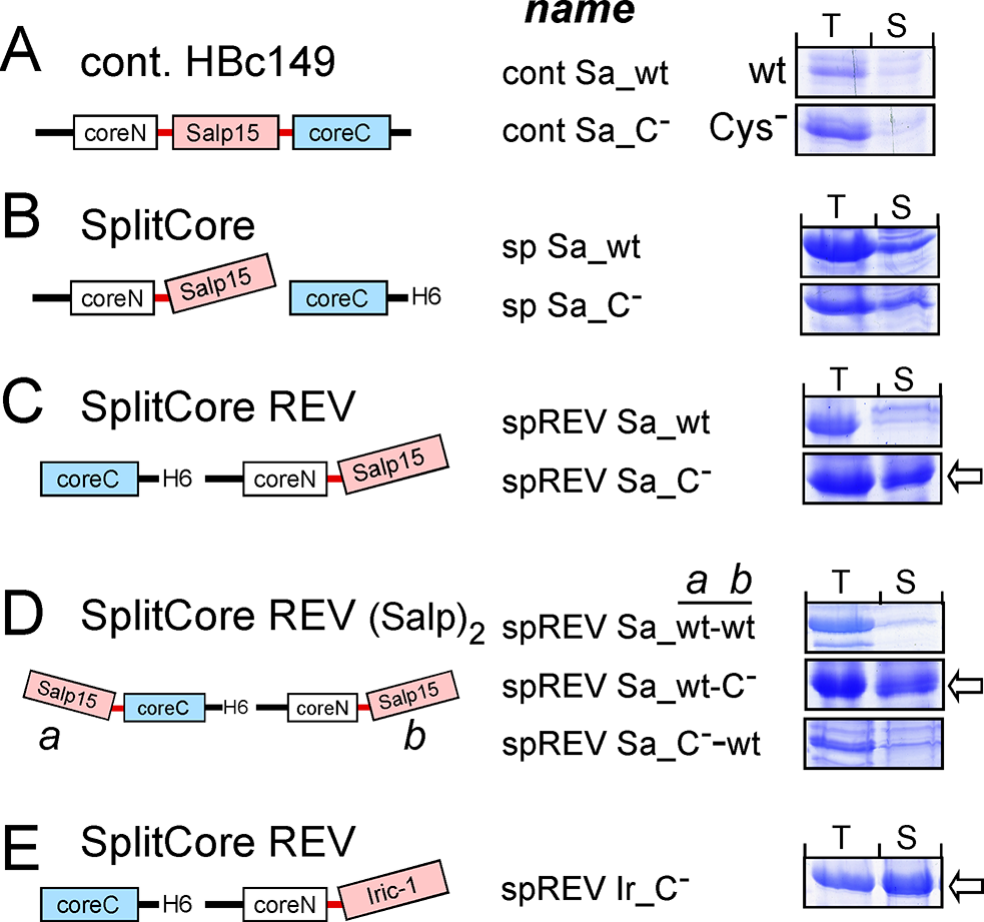
Screening for soluble Salp15 HBc fusions. Cells from induced cultures of BL21*CP cells transformed with the indicated tick protein—HBc fusion constructs were lysed under non-denaturing conditions. Unfractionated total lysate (T) and soluble supernatant (S) after centrifugation were compared by SDS-PAGE. For all SplitCore constructs, the gel section with the tick protein containing large core segment is shown. wt, Wild-type protein; C^-^, Cys-free variant. **(A) Contiguous chain HBc149 with c/e1 inserted Salp15.** The complete gel lanes are shown inS3A Fig. **(B) SplitCore with Salp15 fused to coreN. (C) SplitCore constructs with coreN and coreC ORFs in reverse order (SplitCore REV).** Failure of alternative expression strains to promote higher solubility of the wt-Salp15 fusion is shown in S3B Fig. **(D) SplitCore REV constructs bearing Salp15 on both coreN and coreC. (E) SplitCore REV construct bearing Cys-free Iric-1 on coreN.** The complete gel lanes are shown in S3C Fig. Note the strong positive impact on solubility of replacing the endogenous tick protein cysteines in the SplitCore REV but not the contiguous chain HBc constructs.

For the initial experiments we focused on wt Salp15 and its Cys-free variant and generated pET vectors encoding contiguous chain HBc c/e1 insertions plus several different SplitCore constructs (Fig 5). As expression strain we used *E*. *coli* BL21*CP cells (Stratagene) in which the CP plasmid provides extra tRNAs for rare *E*. *coli* codons, and induction conditions (25°C, 1 mM IPTG as inducer, 16 h induction time) under which HBc fusions with other protein inserts were solubly expressed (e.g. [33]). Expression levels and solubility were scored by non-denaturing lysis, followed by SDS-PAGE of the unfractionated total lysate (T) versus the soluble supernatant (S) after centrifugation.

The contiguous HBc149 c/e1 insertion constructs were well expressed but completely insoluble (Fig 5A; the complete lanes of the SDS-PAGE gel are shown in S3A Fig). Higher solubility was neither achieved by lowering the induction temperature to 18°C, or by reducing the induction time to 8 h or 4 h or the inductor concentration to 100 μM. Also using the cytoplasmic disulfide-formation promoting expression strain T7 SHuffle Express (NEB) or cytoplasmic co-expression of the yeast sulfhydryl oxidase Erv1p plus the disulfide isomerases DsbC or PDI, reportedly improving soluble expression of other Cys-rich proteins in *E*. *coli* [46], had no positive effect. Therefore, we next investigated HBc149 based bicistronic SplitCore expression constructs [31]. Fusing wt or Cys-free Salp15 to the coreN segment present as upstream cistron appeared to enhance solubility to some extent (Fig 5B) yet during subsequent purification attempts the proteins largely precipitated upon dialysis against PBS (data not shown).

A further option unique to the SplitCore system relies on the cap-independent translation mechanism of prokaryotes which allows to reverse the order of the coreN and coreC open reading frames (ORFs) on a bicistronic mRNA (the corresponding constructs are designated with the acronym REV). In normal versus REV constructs the translation initiation sites of each ORF experience a different sequence context which may remedy RNA secondary structures obstructing initiator ATG accessibility (Thi Thai An Nguyen, PhD thesis, University of Freiburg, 2012; [32]).

**Fig 6:**
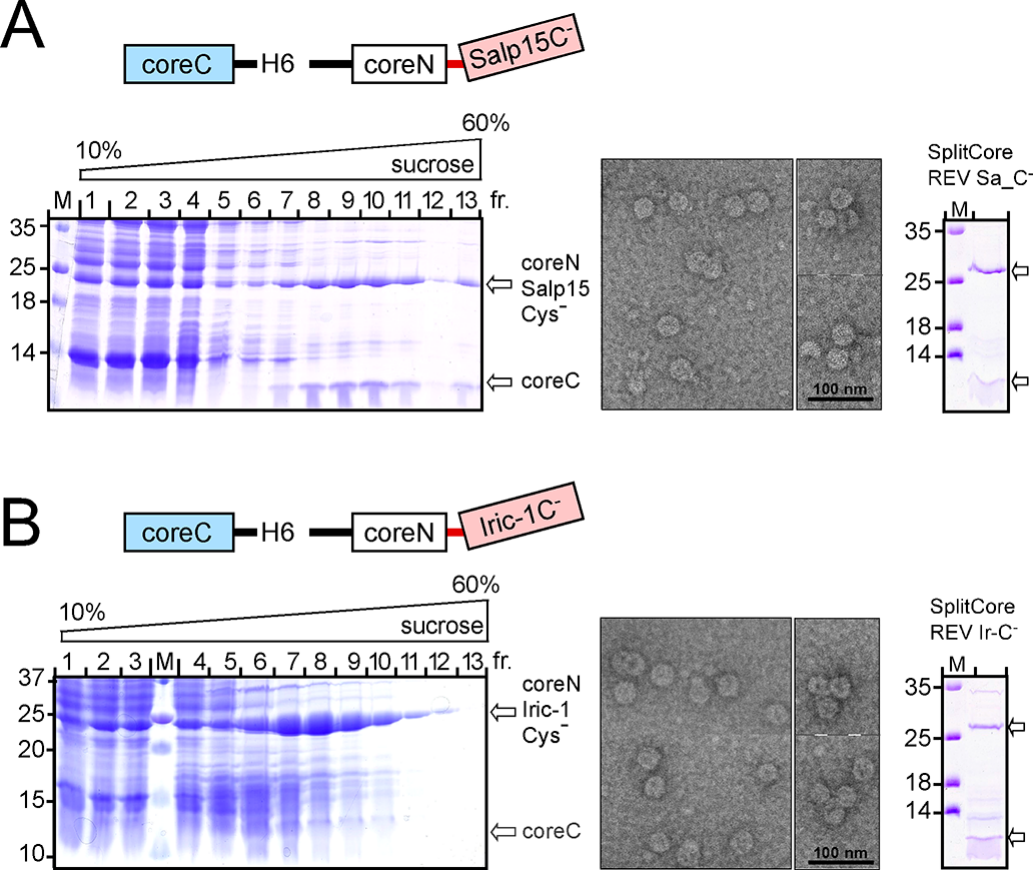
SplitCore REV fusions with Cys-free Salp15 and Iric-1 form regular CLPs. Cleared lysates from bacteria transformed with the indicated SplitCore REV constructs were subjected to sucrose gradient sedimentation and analyzed by SDS-PAGE as in Fig 3B. The positions of the large and small SplitCore fragments is indicated by arrows. **(A) CoreN fusion with Cys-free Salp15.** *Left*: Coomassie Blue stained SDS-PAGE gel. *Middle*: Negative staining EM of material from fraction 8. Sometimes aggregates of several particles were observed (smaller fields on the right). *Right*: SDS-PAGE analysis of the final preparation used in the subsequent immunization experiments. **(B) CoreN fusion with Cys-free Iric-1.** Designations of the panels are as in (A). The poor visibility of the coreC segment in the left panel is due to its small size of ~9 kDa; its presence was proven by efficient CLP formation (*middle panel*) and directly by SDS-PAGE analysis of the further purified preparation (*right panel*) obtained by resedimentation of the pooled fractions 6 to 10 from the first gradient.

Reversing the cistron order had no positive impact for the wt Salp15 SplitCore REV construct (spREV Sa_wt) in BL21*Cp cells (Fig 5C, upper panel), and neither in SHexpCP cells, or in Arctic Express cells (Agilent Technologies) induced at 15°C (S3B Fig). However, of the analogous Cys-free construct (spREV Sa_C^-^) more than half remained soluble (Fig 5C, lower panel). Building upon this result, we generated REV constructs bearing two copies of Salp15, either wt or Cys-free (C^-^), one each on coreN and coreC (Fig 5D; designated spREV Sa_a-b, with a and b indicating the type of Salp15 protein on coreC and on coreN, respectively). Of all combinations tested, only construct spREV Sa_wt-C^-^t yielded a well detectable fraction of soluble material (Fig 5D, middle). Upon subsequent sucrose gradient sedimentation, however, most of the protein sedimented far down in the gradient (data not shown), as is typical for aggregates [33]. We therefore focused on construct spREV Sa_C^-^ carrying a single copy of Cys-free Salp15 on coreN. As shown in Fig 6A (left panel), most of the two protein fragments co-accumulated in fractions 8 to 10, with only minor amounts in the bottom fraction. To confirm proper CLP formation, material from fraction 8 was analyzed by negative stain EM which revealed the presence of regular spherical particles (Fig 6A, middle panel), mostly with diameters of around 40 nm. An SDS-PAGE analysis of the CLP preparation used in the subsequent immunization experiments is shown in Fig 6A (right panel).

Together, these data indicated that only the combination of the SplitCore REV carrier with Cys-free Salp15 was suitable for CLP production.

Of the analogous Iric-1 constructs (spREV Ir_wt and spREV Ir_C^-^) the wt protein was again nearly insoluble (not shown) but a large fraction the Cys-free construct was soluble (Fig 5E, S3C Fig). Upon sucrose gradient sedimentation, it accumulated in the CLP-typical fractions (Fig 6B, left panel). On the particular SDS-PAGE gel shown, the small coreC segment was not clearly detectable; however, the spherical particles visible in negative staining EM (Fig 6B, middle panel) could not form in its absence [47,48]; furthermore, after resedimentation of the pooled material from fractions 7 to 10 the small coreC segment became directly detectable (Fig 6B, right panel). Altogether, these experiments provided sufficient amounts of CLPs displaying Cys-free Salp15 and Iric-1 for the subsequent immunization experiments.

### High level anti-wild-type tick protein antibodies in mice immunized with CLPs presenting Cys-free Salp15 and Iric-1

Given the immunosuppressive activity of Salp15 and Iric-1 plus the unknown impact of the Cys>Ser exchanges on their ability to induce wild-type protein specific antibodies we sought to maximize immunogenicity of the CLP preparations. Based on previous results suggesting additive effects of CLP display and adjuvant [49] we first compared the antibody responses evoked by HBc149-Salp15Cys^-^ CLPs administered without versus with MPL. Groups of three mice each were repeatedly immunized with 10 μg/shot of the CLPs, or in the additional presence of MPL. Relative anti-Salp15 titers were determined by ELISA, using recombinant wt H6-Salp15 solubilized from inclusion bodies [10] as test antigen. As shown in S4A Fig, both immunization protocols induced a strong anti-Salp15 response despite the CLPs presenting Cys-free, not wt, Salp15. MPL increased titers by about two-fold and was thus used in all subsequent immunizations.

IS from the CLP plus MPL immunization were also used to estimate the impact of the Cys-exchanges on antigen recognition. To this end ELISAs were performed using DsbA-fused wt versus Cys-free Salp15 as test antigens (S4B Fig). Accordingly, the wt antigen was recognized about two-fold less efficiently than the autologous Cys-free antigen, indicating an only moderate negative effect of the cysteine replacements.

**Fig 7:**
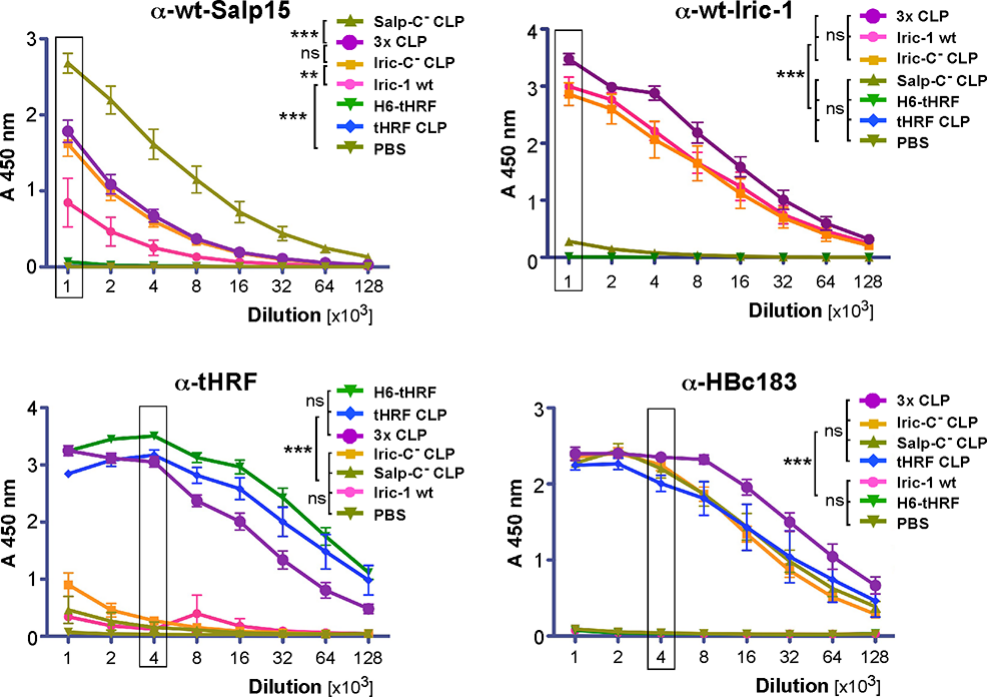
Antibody specificities in mouse IS induced by different tick protein preparations. Groups of 4 to 8 mice were immunized on day 0, 14, 28 and 60 with 10 μg protein per shot in the presence of MPL adjuvant, or with PBS as negative control. Antigens were i) free wt-Iric-1 (Iric-1 wt) released from a DsbA-Iric-1 fusion [10]; ii) H6-tHRF (Fig 3A); iii) spREV Sa_C^-^ CLPs (Salp-C^-^ CLP; Fig 6A); iv) spREV Ir_C^-^ CLPs (Iric-C^-^ CLP; Fig 6B); v) c149-tHRF CLPs (Fig 3B); vi) a 1:1:1 (per weight) mixture of all three CLP preparations (3x CLP). Two-fold serial dilutions from 1:1,000 to 1:128,000 of individual IS obtained on day 67 p.i. were analyzed in duplicate by ELISA. Plots show the mean absorbance at 450 nm ± SEM versus dilution. Significance of differences between groups was assessed at the highest dilution where the most strongly reactive IS per series reached the maximum value (boxed). **(A) Anti-wt-Salp15 response.** Solubilized H6-Salp15 served as test antigen. **(B) Anti-wt-Iric-1 response.** Free wt-Iric-1 released from DsbA-Iric-1 served as test antigen. **(C) Anti-tHRF response.** SEC purified H6-tHRF served as test antigen. **(D) Anti-HBc183 response.** Sucrose gradient purified HBc183 CLPs served as test antigen. Note the strong specific responses to the protein antigens present in the respective vaccines, and the high crossreactivity against wild-type Salp15 and Iric-1 induced by the CLPs presenting their Cys-free variants (see S4B Fig for direct cross-antigenicity).

Next, groups of 8 mice each received 10 μg/shot of HBc149-Salp15Cys^-^ CLPs and HBc149-Iric-1Cys^-^ CLPs (Salp-C^-^ CLP and Iric-C^-^ CLP in Fig 7). Groups of 4 mice each received recombinant wt Iric-1 released from the DsbA fusion [10]; an SDS-PAGE analysis of the preparation used is in shown in S5 Fig. Due to its lower solubility [10], analogously prepared free Salp15 was not available in sufficient quantity and concentration. Further groups of four mice were immunized with H6-tHRF and HBc149-tHRF CLPs (tHRF CLP in Fig 7), or with a combination of all three CLPs (3.3 μg each per shot; 3x CLP in Fig 7). Finally, a control group of four mice were injected with PBS plus MPL. Following the first injection (day 0), immunizations were repeated on day 14, 28, and 60. Serial two-fold dilutions (from 1:1,000 to 1:128,000) of the IS obtained at day 67 were then assessed for target protein specific antibodies by ELISA, using *E*. *coli* derived solubilized H6-Salp15, free wt Iric-1, or H6-tHRF as test antigens. The Iric-1 preparation, also used for immunization, contained some *E*. *coli* antigens, as indicated by the ELISA reactivity of the Iric-1 induced IS (but not any of the CLP-induced IS) with a plate-immobilized crude preparation of an analogously expressed unrelated protein (DsbA-GFP; data not shown). These non-specific reactivities were efficiently quenched by preadsorption with the crude DsbA-GFP preparation. Anti-HBc responses were monitored using immobilized HBc183 CLPs.

As shown in Fig 7A–7C, all IS contained target-specific antibodies, as indicated by the high signals obtained with the IS to immunogens containing the cognate target protein compared to the controls. H6-Salp15 (Fig 7A) reacted most strongly with the IS induced by HBc149-Salp15Cys^-^ CLPs, corroborating the presence of common, Cys-independent epitopes. Signals of about half the strength were also seen with the IS derived from the Iric-1-Cys^-^ CLPs and the combination of all three CLPs, and, less pronouncedly, from free Iric-1. Hence Iric-1 can induce antibodies that are partially cross-reactive with Salp15.

Free Iric-1 (Fig 7B) was well recognized by all IS induced by Iric-1 containing antigens but only very poorly by the IS against Cys-free Salp15 presenting CLPs; this low cross-reactivity was confirmed in subsequent experiments (see below).

The ELISA data for tHRF corroborated the results from the previous immunization in that all IS derived from tHRF-containing immunogens, but not the others, reacted strongly with the immobilized H6-tHRF (Fig 7C).

Lastly, all CLP-derived but not the other IS reacted with immobilized HBc183. Notably, the relative anti-target protein versus anti-carrier responses were higher for tHRF than for Salp15 or Iric-1 as target, as estimated by the IS dilutions giving a half-maximal signal. This might reflect an intrinsically higher immunogenicity of tHRF and/or relate to the fact that the IS to Salp15 and Iric-1, but not tHRF, were raised against the Cys-free variants but tested against the wild-type proteins.

Together, these data showed the ability of IS against CLP-presented Cys-free Salp15 and Iric-1 to specifically recognize their recombinant parental wt antigens, plus a detectable cross-reactivity of the anti-Iric-1-Cys^-^ antibodies with wt Salp15 but not *vice versa*. Notably, in the three-CLP combination one third the amount of each antigen compared to the single CLP immunizations was still able to evoke similar titers of target-specific antibodies.

### *In vitro* assessment of the neutralizing potential of antibodies induced by CLPs presenting Cys-free Salp15 and Iric

Addressing the protective potential of the antibodies induced by our CLP vaccines will require tick challenge and borrelia transmission experiments. To justify such animal experiments, we addressed four parameters *in vitro* that are crucial for a protective activity of the antibodies *in vivo*, namely their ability to recognize glycosylated Salp15, a broad antibody repertoire towards different target protein sequence regions, a potential interference of pertinent antibodies with the interaction of Salp15 and Iric-1 with OspC, and an impact of anti-Salp15 antibodies on complement-mediated killing of Salp15 decorated borreliae.

**Fig 8:**
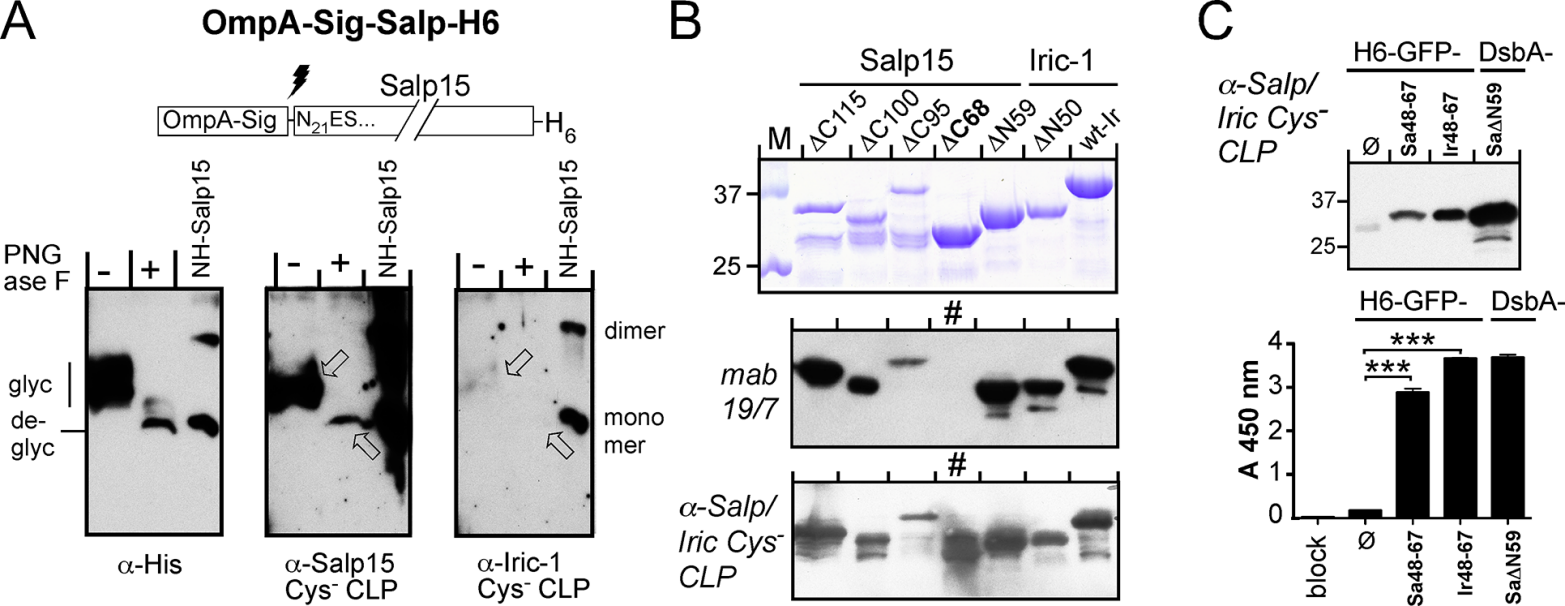
Breadth of antibody reservoirs evoked by CLPs presenting Cys-free Salp15 and Iric-1. **(A) Recognition of secreted glycosylated Salp15.** C terminally His6 tagged Salp15 secreted from HEK 293 cells transfected with an OmpA signal peptide bearing construct (OmpA-Sig-SalpH6) was affinity-captured using Ni-NTA beads. One part was analyzed by immunoblotting without further treatment (-), the other after incubation with PNGase F to remove N-linked glycans (+); the positions of glycosylated (glyc) and deglycosylated (deglyc) Salp15 are indicated. 50 ng of solubilized recombinant H6-Salp15 served as reference. Three parallel blots were individually probed with an anti-His-tag mab, and IS against Cys-free Salp15 or Cys-free Iric-1 CLPs. The anti-Iric-CLP IS showed almost no reaction with secreted Salp15 and reacted at least ten-fold less efficiently with the reference protein. The upper bands in the reference lanes recognized by this IS and also the anti-His-tag mab represent S-S-linked dimers present in the solubilized H6-Salp15 preparation. **(B) Antibodies in IS induced by Cys-free tick protein presenting CLPs recognize epitopes all along the primary sequence of wt Salp15.** *E*. *coli* derived DsbA fusions with truncated Salp15 and Iric-1 [10] served as test antigens. *Top*: Coomassie Blue stained SDS-PAGE. *Middle*: A second SDS-PAGE gel was loaded with one fifth the amount of each protein, blotted, and probed with mab 19/7.4 which recognizes an epitope between residues 83 and 92 of Salp15 that is conserved in Iric-1. Variant Salp15 ΔC68 lacks this epitope and is not recognized (lane marked with #). *Bottom*: Antigens were blotted as in the middle panel but probed with pooled IS (α-Salp/Iric Cys^-^ CLP) from the immunizations with CLPs presenting Cys-free Salp15 and Iric-1. The strong reaction with the DC68 protein demonstrates the presence of epitopes upstream of Salp15 aa 68. **(C) Recognition of the OspC interaction sites on Salp15 and Iric-1.** Recombinant His-tagged GFP (H6-GFP) bearing no extra sequence (ø) or the OspC binding regions (aa 48–67) of Salp15 or Iric-1 [10] was probed with the α-Salp/Iric Cys^-^ CLP IS; DsbA-SaΔN59 protein served as positive control. *Top*: Immunoblot, developed using a secondary anti-mouse PO conjugate and chemiluminescent substrate. *Bottom*: ELISA, using the indicated proteins as immobilized test antigens. Lane "block", blocking solution only. Note the significantly stronger reaction of the IS with GFP bearing the OspC interacting Salp15 and Iric-1 regions compared to GFP only.

Regarding potential epitope shielding by glycans on genuine Salp15, a signal sequence-containing Salp15 with a C teminal His6-tag (Fig 8A) was expressed in human HEK293 cells and affinity-captured from the culture supernatant using Ni-NTA agarose [10]. One part was then incubated with peptide-N-glycosidase F (PNGase F) to remove N-bound glycans, the other part was left untreated. Both samples were then analyzed by SDS-PAGE and immunoblotting, using either a mab against the His-tag, or pooled mouse IS induced by SplitCore Salp15Cys^-^ or Iric-1-Cys- CLPs; on each blot, one lane was loaded with 50 ng of solubilized recombinant H6-Salp15 as a positive control. The anti-His-tag mab detected secreted Salp15-His6 as a smeary band above the 25 kDa marker position that collapsed into one major band upon PNGase treatment that nearly comigrated withrecombinant H6-Salp15 (which was in part present as a dimer, marked by an asterisk in Fig 8A). The weaker signal for the PNGase F treated sample results from a partial loss of material during the enzymatic treatment; as this sample was split into equal aliquots for all three immunoblots, this also holds for the other blots. The Salp15Cys^-^ CLP induced IS reacted well with both the glycosylated and deglycosylated secreted Salp15, and even stronger with the recombinant H6-Salp15 than the anti-His-tag antibody. In contrast, the IS to Iric-1-Cys^-^ CLPs gave barely detectable signals with the secreted Salp15 and also reacted much more weakly, though detectably, with H6-Salp15. Hence the Salp15-Cys^-^ CLP induced antibodies were capable of recognizing near-authentic glycosylated Salp15, whereas only a low-level anti-Salp15 response was evoked by Iric-Cys^-^ CLPs.

To address the breadth of the anti-Salp15 antibody response, we performed immunoblots with a series of DsbA-Salp15 fusions [10] with truncations at the N terminus (SaΔN, followed by position of the last deleted aa) or the C terminus (SaΔC, followed by the position of the first deleted aa). Mab19/7.4, recognizing Salp15 residues 83–91 [10], served as a control that is non-reactive with variant SaΔC68 (Fig 8B, middle panel; lane marked with an asterisk). In contrast to the mab, the IS to Salp15Cys^-^ CLPs gave clear signals with all truncated Salp15 variants including SaΔC68 (Fig 8B, bottom panel). Hence the IS contained antibodies towards multiple epitopes on Salp15.

Regarding interference with OspC binding, we first assessed whether the IS were able to recognize the OspC interaction sites on Salp15 and Iric-1 located between aa 48–67 [10]. As shown in Fig 8C (top panel), GFP fused to these sequences, but not free GFP, gave strong signals in an immunoblot probed with the Cys-free Salp15 and Iric-1 CLP induced IS. These results were confirmed in a non-denaturing ELISA setting, using the GFP fusions, free GFP and DsbA-fused SaΔN59 as immobilized test antigens; wells preincubated exclusively with blocking solution served as negative control. Probing with the CLP-induced IS (Fig 8C, bottom panel) generated strong signals with the tick protein containing samples but not with free GFP or the negative control. Hence the IS contained antibodies recognizing the OspC interaction regions.

**Fig 9:**
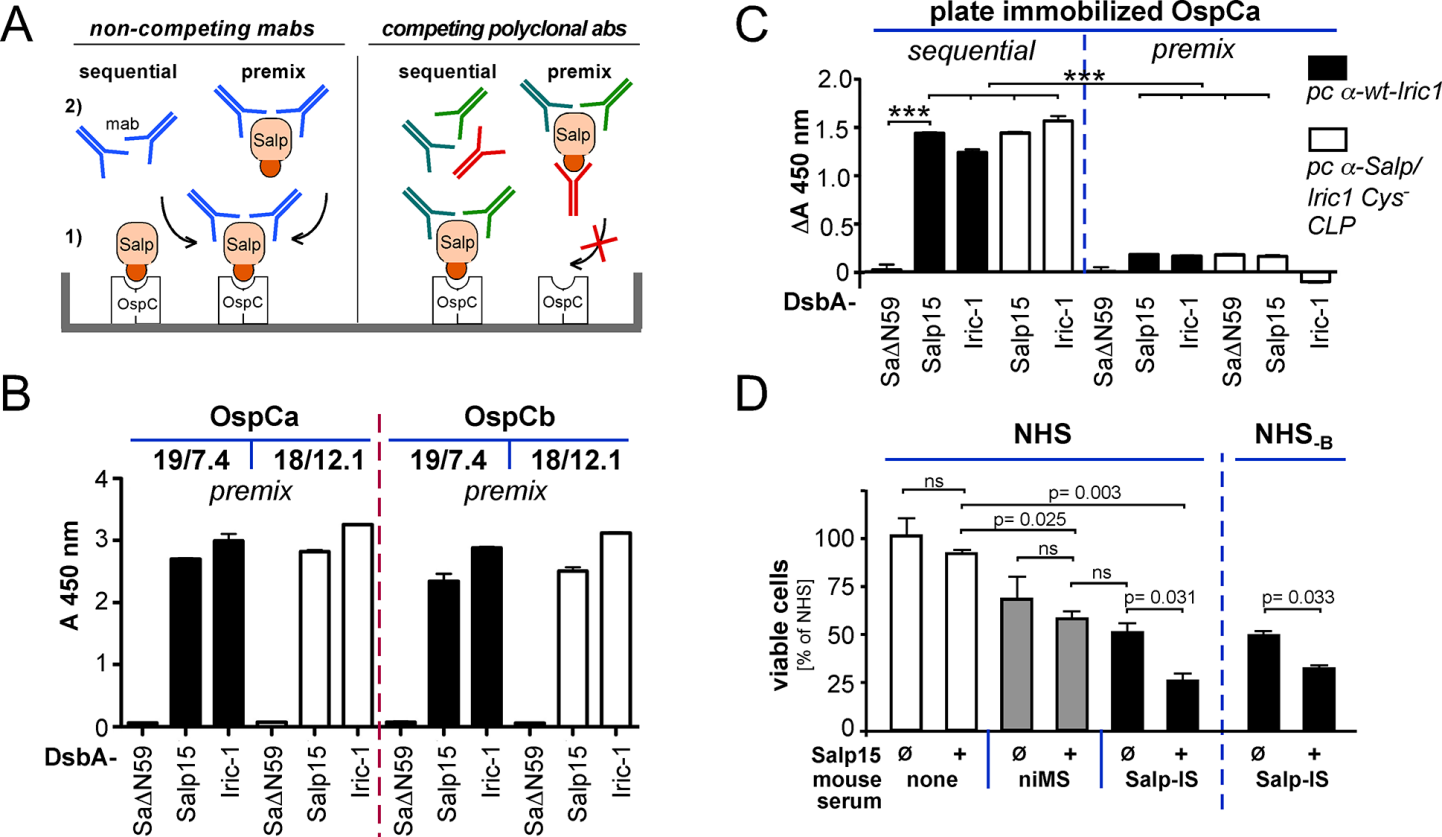
*In vitro* evidence for anti-borrelial activity of vaccination-induced anti-Salp15 antibodies. **(A-C) Interference with the Salp15-OspC interaction. (A) Competitive ELISA scheme.** In the usual sequential procedure (*left*) Salp15 can (1) bind via its interaction region (red) to immobilized OspC. Bound Salp15 can then be detected (2) using anti-Salp15 mabs 19/7.4 and 18/12.1 [10]. As the mab epitopes are outside the OspC binding region, preincubation (premix) of Salp15 with the mabs should not interfere with OspC binding (*left part*). If the vaccination induced polyclonal IS contain antibodies to the OspC binding region (in red), the sequential format should still allow Salp15—OspC binding; however, in the premix format such antibodies would compete with OspC binding. **(B) Assay validation using non-competing mabs.** DsbA-fused Salp15, Iric-1 or non-OspC binding SaΔN59 protein as negative control were premixed with excess mab 19/7.4 or 18/12.1, then the mixtures were added to plate-immobilized *B*. *burgdorferi* OspCa or OspCb. Bound mab complexes were detected using a secondary anti-mouse-PO conjugate. As expected, no interference was observed. **(C) Antibodies in vaccination-induced IS compete with OspC binding.** In the sequential format, the polyclonal IS to wt-Iric-1 (pc α-wt-Iric1) and pooled IS to CLPs presenting Cys-free Salp15 and Iric-1 (pc α-Salp/Iric Cys^-^ CLP) gave strong signals upon OspCa-mediated binding of DsbA-fused wt-Salp15 and wt-Iric-1. In contrast, signals were markedly reduced in the premix format. Comparable results were obtained for OspCb, and *B*. *afzelii* OspC A3 and YU (S6 Fig). **(D) Anti-Salp15 antibodies in the presence of Salp15 enhance killing of borreliae by human complement.** B313 borreliae were preincubated in the absence or presence of recombinant H6-Salp15 with either anti-Salp15-specific mouse IS (Salp-IS) or non-immune mouse serum (niMS), and thereafter in 25% (v/v) normal human serum (NHS). Cell counts were determined in triplicate by a fluorescence-based survival assay [38] and normalized to the mean value obtained in NHS without any additives (set to 100%); error bars denote SEM. Note that the simultaneous presence of both Salp15 and anti-Salp15 antibodies (Salp-IS + Salp) caused a further, significant drop in viability (p = 0.031) compared to niMS ± Salp. Comparable results with factor B depleted NHS (NHS_-B_) confirmed the antibody-dependence of the effect. See text for further controls and experimental details.

Next we modeled a potential interference of the CLP-induced IS with the Salp15—OspC interaction in an ELISA format (Fig 9A) in which antibodies to the OspC binding site on soluble Salp15 proteins would compete with Salp15´s binding to immobilized OspC. To establish the assay, we first used the anti-Salp15 mabs 18/12.1 and 19/7.4 which detect Salp proteins bound to immobilized OspC [10] when added after the Salp-OspC interaction has occurred (Fig 9A, sequential format). To mimic the competition situation, here the mabs were premixed (Fig 9A, premix format) at a 4:1 molar ratio with DsbA-Salp15, DsbA-Iric-1 or variant DsbA-SaΔN59 which does not bind OspC but is still recognized by both mabs. Subsequently, the mixtures were incubated with plate-immobilized *B*. *burgdorferi* OspCa or OspCb. Bound Salp protein-mab complexes were then detected using anti-mouse IgG. As shown in Fig 9B, in this premix format both mabs still gave strong signals with the full-length Salp15 and Iric-1 fusion but not with the OspC binding-incompetent SaΔN59 protein. Hence the mabs did not compete with the Salp protein—OspC interaction, as expected from the distinct location of their epitopes with respect to the OspC binding region [10].

Next the IS to wt-Iric-1 or pooled IS to Cys-free Salp15 and Iric-1 CLPs were analyzed in the sequential versus premix format (Fig 9C), using 100 μl of a 1:100 IS dilution per reaction. This was a ten-fold higher concentration than the highest concentration used for detection of 100 ng/well directly immobilized tick antigen in the titrations shown in Fig 7, aiming to provide excess antibody over the soluble DsbA tick proteins. Nonspecific signals arising from this high IS concentration, seen in preliminary experiments, were quenched by supplementation with *E*. *coli* lysate. Remaining background, as measured by low but detectable signals with the non-OspC binding SaΔN59 protein, was accounted for by subtracting the corresponding absorbances with this protein in the sequential setting from all other values.

As shown for immobilized OspCa protein in Fig 9C, the IS to wt-Iric-1and to Cys-free Salp15 and Iric-1 CLPs gave strong signals in the sequential format which were markedly reduced in the premix format. Comparable data were obtained with immobilized OspCb and with *B*. *afzelii* OspC A3 and OspC YU (S6 Fig). Hence the IS contained antibodies capable of interfering with the Salp protein—OspC interaction.

Lastly, we assessed the impact of the CLP-induced anti-Salp15 antibodies on human complement-mediated killing of OspC-expressing borreliae in the presence versus absence of Salp15. In the naive host, Salp15 reportedly protects the spirochetes from borreliacidal antibodies [7] and reduces deposition of the complement membrane attack complex on the surface of spirochetes [50]. In the presence of preexisting anti-Salp15 antibodies, Salp15 decoration may instead tag the spirochetes for opsonization and complement-mediated killing. We therefore preincubated the OspC expressing serum-sensitive *B*. *burgdorferi* isolate B313 [37,38] in the presence versus absence of recombinant Salp15 with the mouse IS against the combination of tick protein CLPs (Salp-IS) for one h, and then in 25% (v/v) normal human serum (NHS) for another 20 h. Thereafter, cell counts were determined using a previously described fluorescence-based survival assay [38] (Fig 9D). Dependence on anti-Salp15 antibodies was addressed by replacing the Salp-IS by non-immune serum from the PBS plus MPL control mice (niMS), and dependence on active human complement by replacing NHS by heat-inactivated NHS (hiNHS). Viability in hiNHS was not significantly impacted by any of the additives, and was 1.42 ± 0.18-fold higher than in NHS without additives, as expected from a limited complement activation via the alternative pathway [51]. In contrast, different combinations of mouse Salp-IS and Salp15 differentially affected viability in NHS (Fig 9D). Salp15 alone had no significant effect, excluding toxicity of the preparation. Mouse non-immune serum caused an about 35% reduction (probably via borrelia cross-reactive antibodies against general bacterial antigens, e.g. induced by the adjuvant or due to the non-germ-free keeping of the mice) that was not significantly influenced by the additional presence of Salp15. A similar reduction was seen for Salp-IS alone. Importantly, however, here the simultaneous presence of Salp15 caused a further, significant drop in viability (p = 0.031) to about 25% of that in NHS without additives. Very similar results (p = 0.033) were obtained when NHS was replaced by factor B-depleted NHS (NHS_-B_) where complement activation can not occur through the alternative pathway. Hence the excess killing in the presence of anti-Salp15 antibodies plus Salp15 was due to antibody-dependent complement activation.

Together these *in vitro* data imply that immunization-induced antibodies against Salp15 could hamper tick-mediated Borrelia transmission by competing with Salp15 decoration of the bacteria yet also by making already Salp15 decorated spirochetes more vulnerable to human complement.

## Discussion

Based on their Borrelia transmission facilitating activities [52] Salp15 and Iric-1 as well as tHRF are attractive targets for new Lyme disease vaccines [12,53]. Important hurdles are the immunosuppressive properties of the tick proteins and, for Salp15 and Iric-1, the problematic access to sufficient amounts of recombinant antigen.

Here, several steps towards such anti-tick vaccines were achieved, all based on the generation of HBc CLPs displaying full-length tick saliva proteins. Contiguous chain CLPs presenting tHRF were easily accessible, confirmed a clear immune enhancement over free tHRF, and proved its dependence on a physical linkage to the HBc carriers.

Salp15 and Iric-1 were incompatible with c/e1 display on conventional HBc but soluble CLPs presenting Cys-free versions of both proteins were obtained using the SplitCore platform. These CLPs induced strong antibody responses against the recombinant wt tick proteins and glycosylated Salp15, and the functional in vitro data support their having anti-borrelial activities. Not the least, a mixture of CLPs presenting tHRF plus Cys-free Salp15 and Iric-1 induced strong antibody responses against all three targets.

### Protein-chemical implications for CLP design

The distinct protein-chemical properties of the tick proteins used in this study independently reinforce previous concepts on the suitability of whole proteins for display on HBc CLPs [27]. Like other proteins that are well-presentable on contiguous chain HBc CLPs [28], tHRF as such was highly expressed in *E*. *coli* in soluble monomeric form (Fig 3A, S1 Fig). Efficient CLP formation (Fig 3B and 3C) further suggests that its termini are closely juxtaposed, in line with the 3D structure of human TPT1 (pdb accession numbers: 1YZ1, 2HR9) where N and C terminus are only ~8 Å apart. In contrast, free Salp15 and Iric-1 are virtually insoluble when expressed in *E*. *col*, explaining incompatibility with contiguous chain HBc CLP formation (Fig 5A). Although the wt proteins failed to form CLPs also in the SplitCore system (Fig 5B and 5C) exclusively this carrier allowed formation of preparative amounts of CLPs presenting Cys-free Salp15 and Iric-1 (Fig 5C and 5E). Attempts to exploit the higher folding capacity of eukaryotic versus prokaryotic cells [54,55] to access to wt Salp15 presenting SplitCore CLPs via expression in insect cells met with little success (PK and MN; unpublished data) although this approach was functional for CLPs displaying split GFP [10]. Hence at present, generation of SplitCore CLPs presenting Cys-free Salp15 and Iric-1 in *E*. *coli* appears as the only practical procedure, and the associated immunogenicity cost appears limited (see below).

### Immunological implications for whole-protein antigen display on CLPs

In line with our previous studies [29,31,49] the tHRF-CLP immunizations (Fig 4A) confirmed the immune-enhancing effect of displaying a protein on HBc CLPs. Both HBc183- and HBc149-based CLPs induced higher levels of tHRF-specific antibodies than neat H6-tHRF, and for display on HBc183 particles the titer increase was comparable to that by the strong adjuvant MPL-TDM [40]. For the HBc149-based CLPs, this was less pronounced at earlier time points but clearly visible in the IS from the last bleeding (day 147). Notably, the RNA content of the different vaccines correlated with the induced IgG subtypes. Samples with low RNA content (H6-tHRF, H6-tHRF plus MPL and HBc149-tHRF CLPs) evoked predominantly IgG1, indicative of a T_H_2 biased response; in contrast, HBc183-tHRF CLPs containing more RNA induced an excess of IgG2a over IgG1 antibodies, consistent with a T_H_1 biased response (S2 Fig). Hence in line with previous studies on unmodified [41–43] and short peptide presenting HBc CLPs [56,57] the encapsidated bacterial RNA appears able to trigger TLR-7 when present in whole-protein presenting HBc CLPs. This feature may thus be used to direct the immune response into the desired direction.

Wt HBc CLPs coadministered in *trans* had no immune-enhancing impact, regardless of RNA content. This indicates that protein-presenting HBc CLPs are recognized as a single entity by antigen-presenting cells, even though proposedly important specific structural features on the genuine HBc particle surface [58] are covered by the displayed protein. In line, all foreign sequence bearing CLPs induced anti-HBc carrier responses, though to a lesser extent than wt HBc CLPs (Fig 4B).

In sum, these data suggest tHRF presenting HBc CLPs as a straightforwardly accessible source for a vaccine that can induce high-titered anti-tHRF antibodies without need for a strong adjuvant such as CFA [17].

For Salp15 and Iric-1, the lack of efficient recombinant sources for the authentic proteins was overcome at the cost of replacing all endogenous cysteines and lacking glycosylation. However, these surrogates still induced relevant antibodies to the genuine tick proteins. First, the SplitCore tick protein CLPs evoked high titered antibodies against the recombinant wt forms of their target antigens (Fig 7A and 7B). Notably, if expressed as endpoint dilutions (e.g. three-fold above the negative control signal) all titers would reach into the 1:100,000 range or higher. In direct comparison, antibodies against Cys-free Salp15 CLPs reacted still about half as strongy with wt Salp15 as with the autologous Cys-free variant (S4B Fig). Specificity is underscored by the modest crossreactivity with wt Salp15 of the IS against Cys-free Iric-1 CLPs (Fig 7A) which was even weaker in the reverse setting (Fig 7B). The CLP-induced antibodies addressed epitopes all along the Salp protein primary sequence (Fig 8B), including the OspC binding region (Fig 8C). Important for vaccine applications, the anti-Salp15 CLP IS reacted well with glycosylated Salp15 (Fig 8A) indicating that only a fraction of epitopes are shielded by glycan. Furthermore, the IS interfered with the Salp proteins´ interaction with OspC variants from different borrelial species (Fig 9, S6 Fig) and, conversely, they enhanced vulnerability of Salp15 decorated borreliae to human complement-mediated killing (Fig 9D). While this is indirect *in vitro* evidence, the data do suggest that the Cys-free Salp15 or Iric-1 presenting CLPs can indeed induce biologically relevant antibodies.

### Perspectives for anti-tick vaccines

Regarding suitability of Salp15 orthologs and tHRF as vaccine targets, our data revealed both challenges and new perspectives. First, the moderate crossreactivity of antibodies against Iric-1 with Salp15 and more so *vice versa* indicates that targeting an ortholog from one tick species may not, or only modestly, be beneficial against other ticks and their vectored pathogens. Hence a globally useful vaccine would have to comprise more than one Salp15 ortholog, or else regional vaccines adapted to the prevailing tick species need to be considered. For tHRF this is obviated by its high conservation in numerous tick species. The backside is the similarity to human TPT1; that at least some individual mice immunized against tHRF showed signs of crossreactivity with TPT1 will require further evaluation before using tHRF in human vaccines.

Lastly, the complex composition of tick saliva must be borne in mind. Targeting a single factor may not completely defuse the ticks´ anti-host defense armory and, beyond Salp15´s direct fostering of Borrelia transmission, the spirochetes as well as other vectored pathogens likely benefit in various other ways from the specific environment at the tick bite site. This makes a case for combination vaccines. The efficient induction of antibodies against all three target antigens by combining three different CLPs in one vaccine (Fig 7A–7C) provides proof-of-principle for the feasibility of this approach.

Altogether, our results suggest that *in vivo* tick challenge and Borrelia transmission experiments with these novel and preparatively accessible experimental anti-tick vaccines, alone and in combination, are justified and highly worthwhile.

## Supporting Information

S1 Fig Non-tagged tHRF is highly expressed in *E*. *coli* as soluble, monomeric protein. To confirm that the high solubility and monomeric state of H6-tHRF (Fig 3A) were not mediated by the His6-tag, an analogous pET28a2 expression vector as used for H6-tHRF but lacking the His6-encoding sequence was transformed into *E*. *coli* BL21*CP cells. After induction with 1 mM IPTG for 15 h at 25°C cleared cell lysate in TN50 buffer (25 mM Tris/HCl, 50 mM NaCl, pH 7.5) was subjected to ion exchange chromatography on a DEAE Sepharose Fast Flow cartridge connected to an Äkta FPLC system (both GE Healthcare) and developed using a linear NaCl gradient from 50 mM to 560 mM NaCl (*left panel*). Peak fractions 3 to 7 were concentrated by ultrafiltration (Amicon Ultra centrifugal filters, 10 kDa molecular weight cut-off) and subjected to SEC on a Superdex S75 (16/60) column (*right panel*). The peak of the tHRF protein eluted at a volume of ~73 ml; based on a set of marker proteins (BioRad Gelfiltration standard) this corresponds to a molecular mass of ~25 kDa, only slightly higher than the calculated monomeric mass of 20 kDa.
(PDF)

S2 Fig RNA content in tHRF-presenting HBc CLPs affects IgG subtype distribution of tHRF specific antibodies. IS from day 35 post inoculation with c149-tHRF CLPs, c183tHRF CLPs, neat H6-tHRF neat tHRF plus MPL (Fig 4) were analyzed by ELISA, using plates coated with 100 ng per well of H6-tHRF. tHRF-bound IgG was determined using a non-subtype-specific secondary anti-mouse IgG PO-conjugate (total IgG), or conjugates specific for IgG1 or IgG2a. All assays were performed in triplicate on the same plate. The high RNA-content c183-tHRF CLP induced IS contained about three-fold more IgG2a than IgG1, consistent with a T_H_1 biased response. The three other, low RNA content immunogens resulted in excess IgG1 over IgG2a, in line with a T_H_2 biased response.
(PDF)

S3 Fig Assessment of the solubility of different Salp15 and Iric-1 constructs expressed in *E*. *coli*. **(A) Replacement of the seven endogenous Salp15 cysteines by serines does not rescue solubility in the contiguous chain HBc carrier context**. Solubility was assessed by SDS-PAGE and Coomassie Blue staining as described in the legend to Fig 5A, except the whole lanes rather than sections are shown. The asterisk denotes the position of lysozyme (14 kDa) added during the lysis procedure. T, total cell lysate; S, soluble fraction of the lysate after centrifugation. **(B) Lack of positive impact on solubility of wild-type Salp15 presenting SplitCore fusions of different *E*. *coli* expression strains.** The SplitCore REV vector encoding the fusion of wt-Salp15 to coreN (spREV Sa_wt) was transformed into the indicated expression strains. Induction was performed, as usual, for 12–15 h at 22°C in BL21*CP and T7 SHuffle Express CP cells, or at 15°C in Arctic Express cells (Agilent). The latter strain overexpresses a cold-adapted chaperonin; the position of the large subunit GroEL at ~60 kDa is indicated by diamond symbols. T, S are as defined in (A); P refers to the additionally analyzed pellet after centrifugation of the cell lysate. The position of the Salp15-containing SplitCore fragment is indicated by the arrow. Note that the fragment is highly expressed in all strains yet almost absent from the soluble fraction of the lysates. **(C) SplitCore protein with Cys-free Iric-1 fused to coreN expressed from a SplitCore REV vector (spREV Ir_Cys-) is well soluble.** Lysate from BL21*CP cells expressing construct spREV Ir_Cys- was analyzed as in A; the relevant sections of lanes T and S are already shown in Fig 5E. In addition, the soluble fraction S was passed through a 0.22 µm sterile filter (sf). The virtually identical band patterns in samples S and sf confirmed the absence of aggregates.
(PDF)

S4 Fig (A) Enhanced anti-target antibody responses by CLP display plus MPL adjuvanting. Groups of three mice each were vaccinated at days 0, 14, 28 and 126 (open arrowheads) with 10 μg per shot of Cys-free Salp15 presenting SplitCore CLPs (Salp-C- CLP) in the absence (ø) or presence of MPL. Blood samples were taken at the indicated days post inoculation (for day 0 immediately prior to the first immunization) and serial two-fold dilutions of the IS from the individual mice were used to determine the dilution giving a half-maximal ELISA reading, as described in the legend to Fig 4. Solubilized H6-Salp15 protein served as test antigen. Graphs show the mean values ± SEM. The presence of MPL enhanced the Salp15-specific responses by about two-fold (p = 0.0194, two-tailed t-test). (B) Impact of the Cys->Ser replacements in the Salp15 immunization antigen on wild-type Salp15 recognition. Pooled IS (day 49) from the mice immunized with Salp-C- CLPs plus MPL (S4A Fig) were analyzed in triplicate by ELISA, using DsbA-fused wild-type Salp15 or its Cys-free variant; bars show the mean values ± SEM. Recognition of wild-type Salp15 was reduced by about two-fold compared to the autologous Cys-free protein (p = 0.036).
(PDF)

S5 Fig SDS-PAGE analysis of recombinant Iric-1 and H6-tHRF proteins used for immunization. Free wt Iric-1 was obtained by TEV protease cleavage of a bacterially expressed DsbA-Iric-1 fusion protein containing a TEV protease recognition site between DsbA and Iric-1 as previously described (Kolb et al., 2015). An aliquot of the final preparation used for the immunizations shown in Fig 7 was analyzed side-by-side with H6-tHRF (Fig 3A) by SDS-PAGE and Coomassie Blue staining. ***Supplementary reference*:** Kolb P, Vorreiter J, Habicht J, Bentrop D, Wallich R, Nassal M. Soluble cysteine-rich tick saliva proteins Salp15 and Iric-1 from E. coli. FEBS Open Bio. 2015; 5: 42–55.
(PDF)

S6 Fig Vaccination-induced IS to Salp15 and Iric-1 can compete with tick protein binding to different types of OspC. The competitive ELISAs were performed analogously to those shown in Fig 9, except that immobilized *B*. *burgdorferi* OspCa was replaced by its variant OspCb, or the *B*. *afzelii* isolates OspC A3 and OspC YU as indicated on the top of each graph. Less efficient binding of soluble Salp15 and Iric-1 to the *B*. *afzelii* versus *B*. *burgdorferi* OspC proteins in the sequential assay format (*left part of the graphs*) confirmed previous data obtained with the anti-Salp15 mabs 18/12.1 and 19/7.4 (Kolb et al., 2015). Importantly, however, switching to the premix format (*right part of the graphs*) markedly reduced the amounts of bound Salp15 or Iric-1 for all OspC proteins. This implies that antibodies to Salp15 and Iric-1 could reduce transmission of more than one borrelia species. ***Supplementary reference*:** Kolb P, Vorreiter J, Habicht J, Bentrop D, Wallich R, Nassal M. Soluble cysteine-rich tick saliva proteins Salp15 and Iric-1 from E. coli. FEBS Open Bio. 2015; 5: 42–55.
(PDF)
