# Supplementary material for: Whole-Chain Tick Saliva Proteins Presented on Hepatitis B Virus Capsid-Like Particles Induce High-Titered Antibodies with Neutralizing Potential
Source: PLoS One. 2015 Sep 9;10(9):e0136180. doi: 10.1371/journal.pone.0136180 (PMC4564143; doi:10.1371/journal.pone.0136180)
Supplement: S1 Fig — To confirm that the high solubility and monomeric state of H6-tHRF (Fig 3A) were not mediated by the His6-tag, an analogous pET28a2 expression vector as used for H6-tHRF but lacking the His6-encoding sequence was transformed into E. coli BL21*CP cells. After induction with 1 mM IPTG for 15 h at 25°C cleared cell lysate in TN50 buffer (25 mM Tris/HCl, 50 mM NaCl, pH 7.5) was subjected to ion exchange chromatography on a DEAE Sepharose Fast Flow cartridge connected to an Äkta FPLC system (both GE Healthcare) and developed using a linear NaCl gradient from 50 mM to 560 mM NaCl (left panel). Peak fractions 3 to 7 were concentrated by ultrafiltration (Amicon Ultra centrifugal filters, 10 kDa molecular weight cut-off) and subjected to SEC on a Superdex S75 (16/60) column (right panel). The peak of the tHRF protein eluted at a volume of ~73 ml; based on a set of marker proteins (BioRad Gelfiltration standard) this corresponds to a molecular mass of ~25 kDa, only slightly higher than the calculated monomeric mass of 20 kDa. (PDF) [file pone.0136180.s001.pdf]

S1 Fig.

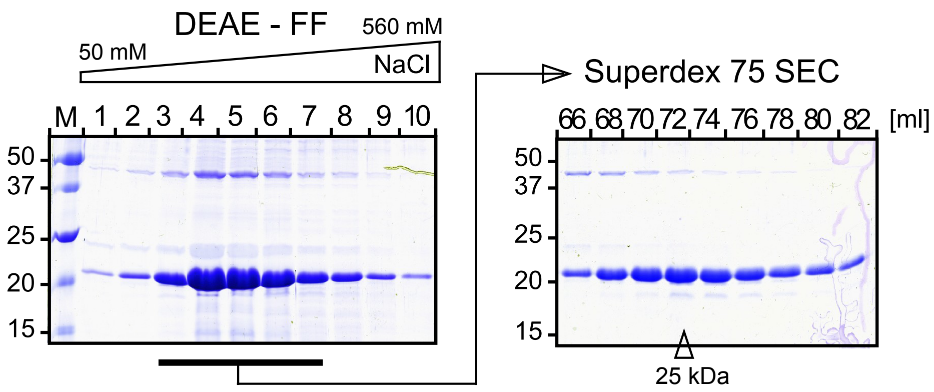

S1 Fig. Non-tagged tHRF is highly expressed in *E. coli* as soluble, monomeric protein. To confirm that the high solubility and monomeric state of H6-tHRF (Fig. 3A) were not mediated by the His6-tag, an analogous pET28a2 expression vector as used for H6-tHRF but lacking the His6-encoding sequence was transformed into *E. coli* BL21*CP cells. After induction with 1 mM IPTG for 15 h at 25°C the soluble fraction of the cell lysate in TN50 buffer (25 mM Tris/HCl, 50 mM NaCl, pH 7.5) was subjected to ion exchange chromatography on a DEAE Sepharose Fast Flow cartridge connected to an Äkta FPLC system (both GE Healthcare) and developed using a linear NaCl gradient from 50 mM to 560 mM NaCl (*left panel*). Peak fractions 3 to 7 were concentrated by ultrafiltration (Amicon Ultra centrifugal filters, 10 kDa molecular weight cut-off) and subjected to SEC on a Superdex S75 (16/60) column (*right panel*). The peak of the tHRF protein eluted at a volume of ~73 ml; based on a set of marker proteins (BioRad Gelfiltration standard) this corresponds to a molecular mass of ~25 kDa, only slightly higher than the calculated monomeric mass of 20 kDa.
