# Supplementary material for: Whole-Chain Tick Saliva Proteins Presented on Hepatitis B Virus Capsid-Like Particles Induce High-Titered Antibodies with Neutralizing Potential
Source: PLoS One. 2015 Sep 9;10(9):e0136180. doi: 10.1371/journal.pone.0136180 (PMC4564143; doi:10.1371/journal.pone.0136180)
Supplement: S2 Fig — IS from day 35 post inoculation with c149-tHRF CLPs, c183tHRF CLPs, neat H6-tHRF neat tHRF plus MPL (Fig 4) were analyzed by ELISA, using plates coated with 100 ng per well of H6-tHRF. tHRF-bound IgG was determined using a non-subtype-specific secondary anti-mouse IgG PO-conjugate (total IgG), or conjugates specific for IgG1 or IgG2a. All assays were performed in triplicate on the same plate. The high RNA-content c183-tHRF CLP induced IS contained about three-fold more IgG2a than IgG1, consistent with a TH1 biased response. The three other, low RNA content immunogens resulted in excess IgG1 over IgG2a, in line with a TH2 biased response. (PDF) [file pone.0136180.s002.pdf]

S2 Fig.

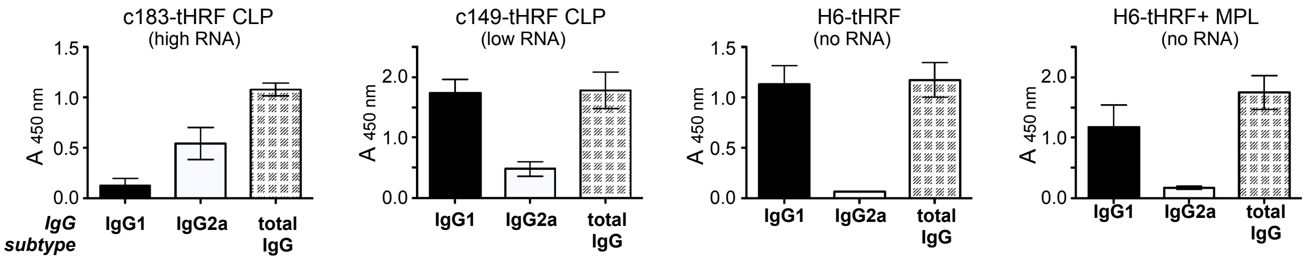

S2 Fig. RNA content in tHRF-presenting HBc CLPs affects IgG subtype distribution of tHRF specific antibodies. IS from day 35 post inoculation with c149-tHRF CLPs, c183-tHRF CLPs, neat H6-tHRF neat tHRF plus MPL (Fig. 4) were analyzed by ELISA, using plates coated with 100 ng per well of H6-tHRF. tHRF-bound IgG was determined using a non-subtype-specific secondary anti-mouse IgG PO-conjugate (total IgG), or conjugates specific for IgG1 or IgG2a. All assays were performed in triplicate on the same plate. The high RNA-content c183-tHRF CLP induced IS contained about three-fold more IgG2a than IgG1, consistent with an TH1 biased response. The three other, low RNA content immunogens resulted in excess IgG1 over IgG2a, in line with a TH2 biased response.
