# Supplementary material for: Whole-Chain Tick Saliva Proteins Presented on Hepatitis B Virus Capsid-Like Particles Induce High-Titered Antibodies with Neutralizing Potential
Source: PLoS One. 2015 Sep 9;10(9):e0136180. doi: 10.1371/journal.pone.0136180 (PMC4564143; doi:10.1371/journal.pone.0136180)
Supplement: S3 Fig — (A) Replacement of the seven endogenous Salp15 cysteines by serines does not rescue solubility in the contiguous chain HBc carrier context. Solubility was assessed by SDS-PAGE and Coomassie Blue staining as described in the legend to Fig 5A, except the whole lanes rather than sections are shown. The asterisk denotes the position of lysozyme (14 kDa) added during the lysis procedure. T, total cell lysate; S, soluble fraction of the lysate after centrifugation. (B) Lack of positive impact on solubility of wild-type Salp15 presenting SplitCore fusions of different E. coli expression strains. The SplitCore REV vector encoding the fusion of wt-Salp15 to coreN (spREV Sa_wt) was transformed into the indicated expression strains. Induction was performed, as usual, for 12–15 h at 22°C in BL21*CP and T7 SHuffle Express CP cells, or at 15°C in Arctic Express cells (Agilent). The latter strain overexpresses a cold-adapted chaperonin; the position of the large subunit GroEL at ~60 kDa is indicated by diamond symbols. T, S are as defined in (A); P refers to the additionally analyzed pellet after centrifugation of the cell lysate. The position of the Salp15-containing SplitCore fragment is indicated by the arrow. Note that the fragment is highly expressed in all strains yet almost absent from the soluble fraction of the lysates. (C) SplitCore protein with Cys-free Iric-1 fused to coreN expressed from a SplitCore REV vector (spREV Ir_Cys-) is well soluble. Lysate from BL21*CP cells expressing construct spREV Ir_Cys- was analyzed as in A; the relevant sections of lanes T and S are already shown in Fig 5E. In addition, the soluble fraction S was passed through a 0.22 µm sterile filter (sf). The virtually identical band patterns in samples S and sf confirmed the absence of aggregates. (PDF) [file pone.0136180.s003.pdf]

S3 Fig.

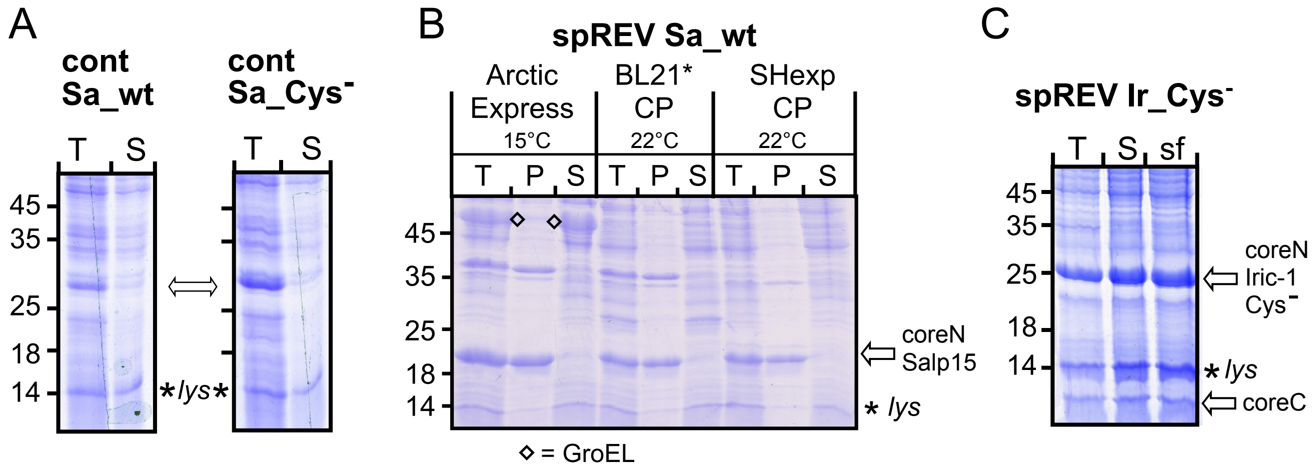

S3 Fig. Assessment of the solubility of different Salp15 and Iric-1 constructs expressed in *E. coli*.
(A) Replacement of the seven endogenous Salp15 cysteines by serines does not rescue solubility in the contiguous chain HBc carrier context. Solubility was assessed by SDS-PAGE and Coomassie Blue staining as described in the legend to Fig. 5A, except the whole lanes rather than sections are shown. The asterisk denotes the position of lysozyme (14 kDa) added during the lysis procedure. T, total cell lysate; S, soluble fraction of the lysate after centrifugation. **(B) Lack of positive impact on solubility of wild-type Salp15 presenting SplitCore fusions of different *E. coli* expression strains.** The SplitCore REV vector encoding the fusion of wt-Salp15 to coreN (spREV Sa_wt) was transformed into the indicated expression strains. Induction was performed, as usual, for 12 - 15 h at 22°C in BL21*CP and T7 SHuffle Express CP cells, or at 15°C in Arctic Express cells (Agilent). The latter strain overexpresses a cold-adapted chaperonin; the position of the large subunit GroEL at ~60 kDa is indicated by diamond symbols. T, S are as defined in (A); P refers to the additionally analyzed pellet after centrifugation of the cell lysate. The position of the Salp15-containing SplitCore fragment is indicated by the arrow. Note that the fragment is highly expressed in all strains yet almost absent from the soluble fraction of the lysates. **(C) SplitCore protein with Cys-free Iric-1 fused to coreN expressed from a SplitCore REV vector (spREV Ir_Cys⁻) is well soluble.** Lysate from BL21*CP cells expressing construct spREV Ir_Cys⁻ was analyzed as in A; the relevant sections of lanes T and S are already shown in Fig. 5E. In addition, the soluble fraction S was passed through a 0.22 µm sterile filter (sf). The virtually identical band patterns in samples S and sf confirmed the absence of aggregates.
