# Supplementary material for: Whole-Chain Tick Saliva Proteins Presented on Hepatitis B Virus Capsid-Like Particles Induce High-Titered Antibodies with Neutralizing Potential
Source: PLoS One. 2015 Sep 9;10(9):e0136180. doi: 10.1371/journal.pone.0136180 (PMC4564143; doi:10.1371/journal.pone.0136180)
Supplement: S4 Fig — Pooled IS (day 49) from the mice immunized with Salp-C- CLPs plus MPL (S4A Fig) were analyzed in triplicate by ELISA, using DsbA-fused wild-type Salp15 or its Cys-free variant; bars show the mean values ± SEM. Recognition of wild-type Salp15 was reduced by about two-fold compared to the autologous Cys-free protein (p = 0.036). (PDF) [file pone.0136180.s004.pdf]

S4 Fig.

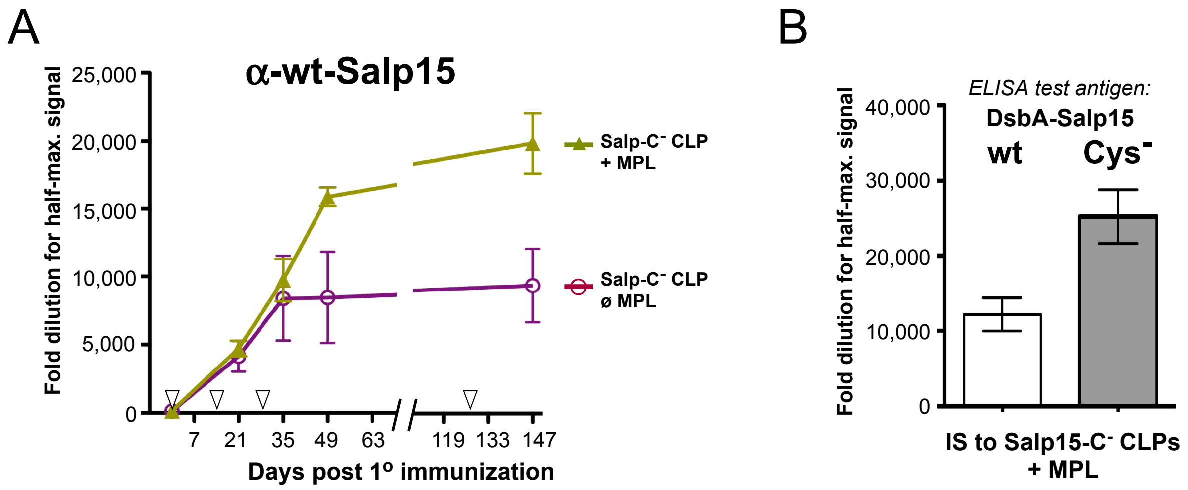

S4 Fig. (A) Enhanced anti-target antibody responses by CLP display plus MPL adjuvanting. Groups of three mice each were vaccinated at days 0, 14, 28 and 126 (open arrowheads) with 10 µg per shot of Cys-free Salp15 presenting SplitCore CLPs (Salp-C$^-$ CLP) in the absence (ø) or presence of MPL. Blood samples were taken at the indicated days post inoculation (for day 0 immediately prior to the first immunization) and serial two-fold dilutions of the IS from the individual mice were used to determine the dilution giving a half-maximal ELISA reading, as described in the legend to Fig. 4. Solubilized H6-Salp15 protein served as test antigen. Graphs show the mean values ± SEM. The presence of MPL enhanced the Salp15-specific responses by about two-fold (p=0.0194, two-tailed t-test). **(B) Impact of the Cys->Ser replacements in the Salp15 immunization antigen on wild-type Salp15 recognition.** Pooled IS (day 49) from the mice immunized with Salp-C$^-$ CLPs plus MPL (S3 Figure A) were analyzed in triplicate by ELISA, using DsbA-fused wild-type Salp15 or the Cys-free variant; bars show the mean values ± SEM. Recognition of wild-type Salp15 was reduced by about two-fold compared to the autologous Cys-free protein (p=0.036).
