# Supplementary material for: Whole-Chain Tick Saliva Proteins Presented on Hepatitis B Virus Capsid-Like Particles Induce High-Titered Antibodies with Neutralizing Potential
Source: PLoS One. 2015 Sep 9;10(9):e0136180. doi: 10.1371/journal.pone.0136180 (PMC4564143; doi:10.1371/journal.pone.0136180)
Supplement: S5 Fig — Free wt Iric-1 was obtained by TEV protease cleavage of a bacterially expressed DsbA-Iric-1 fusion protein containing a TEV protease recognition site between DsbA and Iric-1 as previously described (Kolb et al., 2015). An aliquot of the final preparation used for the immunizations shown in Fig 7 was analyzed side-by-side with H6-tHRF (Fig 3A) by SDS-PAGE and Coomassie Blue staining. Supplementary reference: Kolb P, Vorreiter J, Habicht J, Bentrop D, Wallich R, Nassal M. Soluble cysteine-rich tick saliva proteins Salp15 and Iric-1 from E. coli. FEBS Open Bio. 2015; 5: 42–55. (PDF) [file pone.0136180.s005.pdf]

S5 Fig.

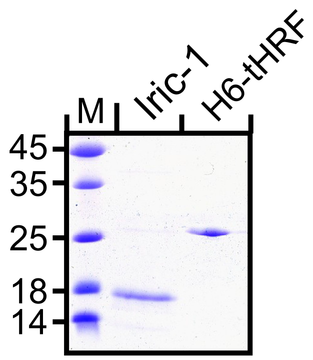

S5 Fig. SDS-PAGE analysis of recombinant Iric-1 and H6-tHRF proteins used for immunization. Free wt Iric-1 was obtained by TEV protease cleavage of a bacterially expressed DsbA-Iric-1 fusion protein containing a TEV protease recognition site between DsbA and Iric-1 as previously described (Kolb et al., 2015). An aliquot of the final preparation used for the immunizations shown in Fig. 7 was analyzed side-by-side with H6-tHRF (Fig. 3A) by SDS-PAGE and Coomassie Blue staining.

Supplementary reference:

Kolb P, Vorreiter J, Habicht J, Bentrop D, Wallich R, Nassal M. Soluble cysteine-rich tick saliva proteins Salp15 and Iric-1 from E. coli. FEBS Open Bio. 2015; 5: 42-55.
