# Supplementary material for: Whole-Chain Tick Saliva Proteins Presented on Hepatitis B Virus Capsid-Like Particles Induce High-Titered Antibodies with Neutralizing Potential
Source: PLoS One. 2015 Sep 9;10(9):e0136180. doi: 10.1371/journal.pone.0136180 (PMC4564143; doi:10.1371/journal.pone.0136180)
Supplement: S6 Fig — The competitive ELISAs were performed analogously to those shown in Fig 9, except that immobilized B. burgdorferi OspCa was replaced by its variant OspCb, or the B. afzelii isolates OspC A3 and OspC YU as indicated on the top of each graph. Less efficient binding of soluble Salp15 and Iric-1 to the B. afzelii versus B. burgdorferi OspC proteins in the sequential assay format (left part of the graphs) confirmed previous data obtained with the anti-Salp15 mabs 18/12.1 and 19/7.4 (Kolb et al., 2015). Importantly, however, switching to the premix format (right part of the graphs) markedly reduced the amounts of bound Salp15 or Iric-1 for all OspC proteins. This implies that antibodies to Salp15 and Iric-1 could reduce transmission of more than one borrelia species. Supplementary reference: Kolb P, Vorreiter J, Habicht J, Bentrop D, Wallich R, Nassal M. Soluble cysteine-rich tick saliva proteins Salp15 and Iric-1 from E. coli. FEBS Open Bio. 2015; 5: 42–55. (PDF) [file pone.0136180.s006.pdf]

S6 Fig.

B. burgdorferi OspCb

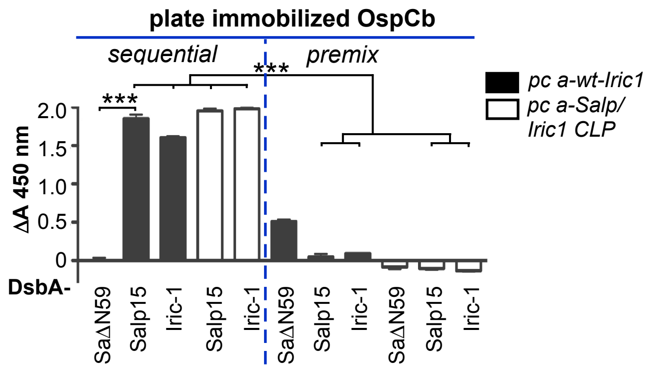

B. afzelii OspCs

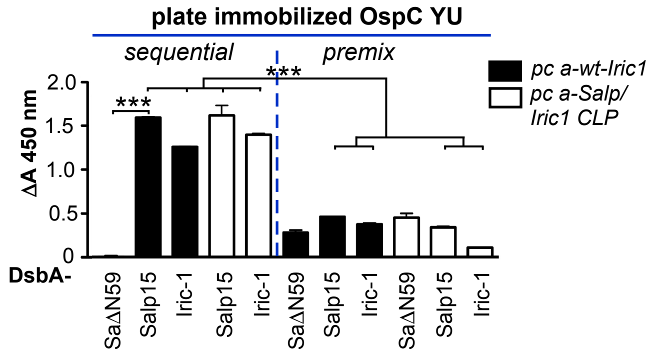

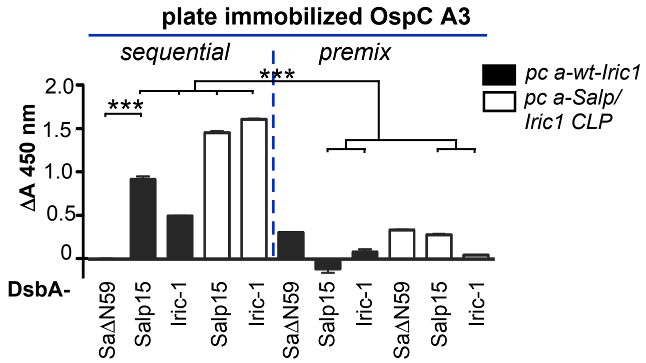

S6 Fig. Vaccination-induced IS to Salp15 and Iric-1 can compete with tick protein binding to different types of OspC. The competitive ELISAs were performed analogously to those shown in Fig. 9, except that immobilized *B. burgdorferi* OspCa was replaced by its variant OspCb, or the *B. afzelii* isolates OspC A3 and OspC YU, as indicated on the top of each graph. Less efficient binding of soluble Salp15 and Iric-1 to the *B. afzelii* versus *B. burgdorferi* OspC proteins in the sequential assay format (*left part of the graphs*) confirmed previous data obtained with the anti-Salp15 mAbs 18/12.1 and 19/7.4 (Kolb et al., 2015). Importantly, however, switching to the premix format (*right part of the graphs*) markedly reduced the amounts of bound Salp15 or Iric-1 for all OspC proteins. This implies that antibodies to Salp15 and Iric-1 could reduce transmission of different borrelia species. Error bars indicate mean values ± SEM (n=3), significance levels (***, p<0.001) refer to comparison of the same tick protein in sequential vs. premix format.

Supplementary reference:

Kolb P, Vorreiter J, Habicht J, Bentrop D, Wallich R, Nassal M. Soluble cysteine-rich tick saliva proteins Salp15 and Iric-1 from E. coli. FEBS Open Bio. 2015; 5: 42-55.
